# Deracemization By Simultaneous Bio-oxidative Kinetic Resolution and Stereoinversion**

**DOI:** 10.1002/anie.201400027

**Published:** 2014-02-24

**Authors:** Joerg H Schrittwieser, Bas Groenendaal, Verena Resch, Diego Ghislieri, Silvia Wallner, Eva-Maria Fischereder, Elisabeth Fuchs, Barbara Grischek, Johann H Sattler, Peter Macheroux, Nicholas J Turner, Wolfgang Kroutil

**Affiliations:** Institut für Chemie, Organische und Bioorganische Chemie, Karl-Franzens-Universität GrazHeinrichstrasse 28, A-8010 Graz (Austria); School of Chemistry, University of Manchester, Manchester Institute of Biotechnology131 Princess Street, Manchester, M1 7DN (UK); Institut für Biochemie, Technische Universität GrazPetersgasse 12, 8010 Graz (Austria)

**Keywords:** alkaloids, asymmetric synthesis, C=C coupling, deracemization, enzyme catalysis, simultaneous cascades

## Abstract

Deracemization, that is, the transformation of a racemate into a single product enantiomer with theoretically 100 % conversion and 100 % *ee*, is an appealing but also challenging option for asymmetric synthesis. Herein a novel chemo-enzymatic deracemization concept by a cascade is described: the pathway involves two enantioselective oxidation steps and one non-stereoselective reduction step, enabling stereoinversion and a simultaneous kinetic resolution. The concept was exemplified for the transformation of *rac*-benzylisoquinolines to optically pure (*S*)-berbines. The racemic substrates were transformed to optically pure products (*ee*>97 %) with up to 98 % conversion and up to 88 % yield of isolated product.

Several techniques have been developed to overcome the limitation of a kinetic resolution (50 % maximum yield), such as dynamic kinetic resolution (DKR),[1] dynamic kinetic asymmetric transformation (DYKAT),[2] stereoinversion,[3,4] or enantioconvergent processes (Scheme 1).[5,6] These techniques allow the process of deracemization to be achieved, that is, conversion of a racemate into an optically pure product with theoretically 100 % yield and 100 % *ee*.

**Scheme 1:**
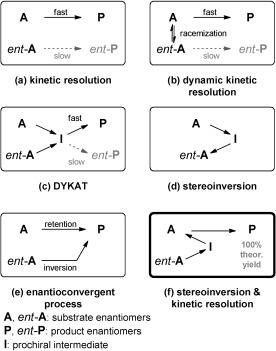
a) Kinetic resolution, b)–e) basic strategies for deracemization, and f) the designed strategy to combine stereoinversion and kinetic resolution.

The berberine bridge enzyme (BBE) catalyzes the enantioselective transformation of 1-benzyl-1,2,3,4-tetrahydroisoquinolines to yield berbines by aerobic C=H activation at the *N*-methyl group, forming a new intramolecular C=C bond (Scheme 2). This reaction has been shown to be suitable for the preparation of various novel optically pure berbines.[7] As the enzyme transforms only one substrate enantiomer, this reaction is a kinetic resolution.

**Scheme 2:**
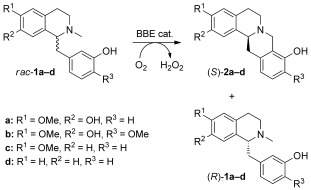
Kinetic resolution by enantioselective oxidative C=C bond formation catalyzed by the berberine bridge enzyme (BBE).

To overcome the limitation of BBE-catalyzed kinetic resolution, we first envisioned to introduce racemization of the substrate enantiomers to set up a DKR. However, this strategy proved problematic for the compounds of interest: Among the published procedures for amine racemization, which rely on homogeneous[8] or heterogeneous[9] metal catalysis, or thiyl radical-mediated reversible H-abstraction,[10] no method was suitable to act exclusively on the benzylisoquinoline substrates whilst leaving the berbine products untouched. For example, Shvo’s diruthenium complex[11] racemized benzylisoquinolines and berbines alike[7a] when employed under previously described reaction conditions.[8g] Raney nickel led to degradation of the complex alkaloids, whereas palladium on charcoal did not show any detectable racemization activity at all.

As a chemical racemization process could not be realized and because no suitable enzyme for racemization of these substrates is known,[12] an alternative was required. However, neither a DYKAT nor an enantioconvergent process seemed feasible. A stereoinversion is also unsuitable, as in this case the product structure is identical to the substrate except for its optical purity. Consequently, we designed a strategy wherein the concept of stereoinversion is combined with kinetic resolution (Scheme 1 f). Generally speaking, stereoinversion of the enantiomer *ent*-**A** will give its mirror image **A**, which will be transformed further in the kinetic resolution to yield exclusively product **P** in theoretically 100 % yield. In the present case, this strategy should allow the conversion of both substrate enantiomers of benzylisoquinolines *rac*-**1** into optically pure berbines (*S*)-**2**.

Stereoinversion by cyclic deracemization of various amines has been described employing a monoamine oxidase (MAO) in combination with an achiral reducing agent.[3] Unfortunately, all established MAOs[3] were unsuitable for substrates **1 a**–**d**. However, a recently developed variant of the MAO from *Aspergillus niger* (MAO-N variant D11) has been reported to oxidize 1-phenyl-1,2,3,4-tetrahydroisoquinoline with high (*R*)-selectivity.[13] The combination of this biocatalytic oxidation with non-stereoselective reduction of the resulting imine by ammonia–borane afforded the (*S*)-enantiomer (*ee*=98 %) from the racemate.

Testing the MAO-N D11 variant for the oxidation of substrates **1 a**–**d** showed that they were indeed accepted (Figure 1). Additionally and importantly, the desired products including **2 c** and **2 d** were not oxidized, which is the prerequisite for their stereochemical stability under the conditions of a final simultaneous cascade process. Chiral analysis of the MAO-N catalyzed oxidations revealed that **1 a**–**c** were converted with excellent enantioselectivity (*E*>200), transforming essentially only the (*R*)-enantiomer, while the enantioselectivity was low for **1 d** (*E*=6.5). Consequently, **1 d** was not investigated further.

**Figure 1:**
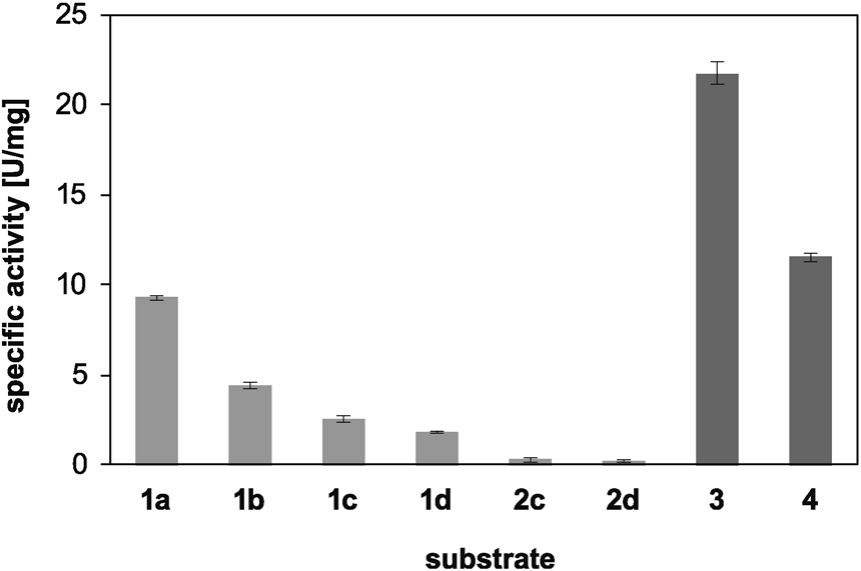
Results of substrate screening with MAO-N variant D11. Activity (criterion: mean> 5 standard deviations) was found for substrates 1 a–d (light gray), as well as the positive controls *rac*-phenylethylamine 3 and *rac*-crispine A 4 (both dark gray). Error ranges represent standard deviations of triplicate experiments.

Previous studies have found that BBE is inhibited by reducing agents,[14] as we confirmed for the ammonia–borane complex, which is most commonly employed in combination with MAO-N. We hypothesized that more bulky or less water-soluble boranes would be more compatible with BBE, as they would be prevented from entering the active site of the enzyme. Consequently, *t*BuNH_2_⋅BH_3_, Me_3_N⋅BH_3_, and morpholine⋅BH_3_ were tested as alternative reductants and proved more suitable for our system (Supporting Information, Figure S1).

Comparing the Me_3_N⋅BH_3_ and morpholine⋅BH_3_ complex with NH_3_⋅BH_3_ in the stereoinversion (MAO), it turned out that the former two actually performed better than the commonly employed reducing agent NH_3_⋅BH_3_, whereby morpholine⋅BH_3_ worked best (Supporting Information, Figure S2).

After these prerequisite investigations, a stepwise one-pot procedure was tested first (Scheme 3); thus, the stereoinversion of substrates *rac*-**1 a**–**c** by the MAO-N/borane-system was run to completion before BBE was added to the reaction mixture. The BBE-catalyzed ring-closure was either carried out after removal of the MAO-N biocatalyst by centrifugation, or BBE was directly added to the reaction mixture.

**Scheme 3:**
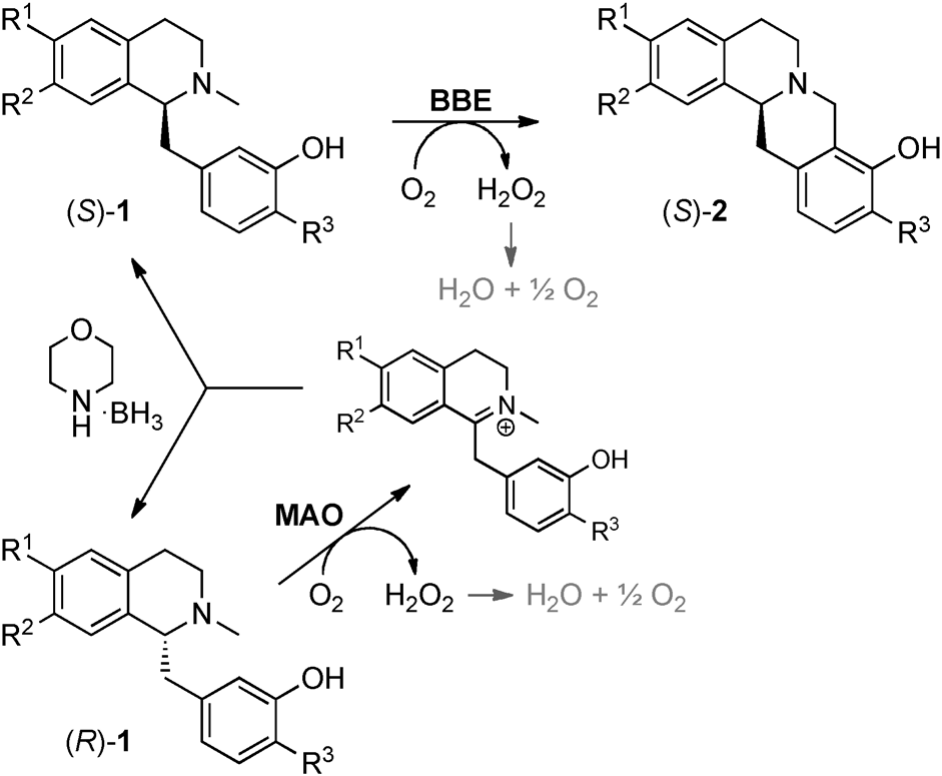
Deracemization of benzylisoquinolines *rac*-1 to berbines (*S*)-2 by a MAO/BBE/borane redox cascade (performed either in a stepwise or a concurrent fashion). MAO=monoamine oxidase MAO-N (variant D11).

The overall transformation of *rac*-**1** to (*S*)-**2** proceeded similarly well in both cases, resulting in high conversions (Table 1). Additionally, all compounds were obtained in enantiomerically pure form (*ee*>97 %, HPLC), which indicated that the enantioselectivity of BBE was not impaired by the extended reaction system.

**Table 1:** Deracemization of benzylisoquinolines *rac*-1 to berbines (*S*)-2 by a stepwise transformation using MAO in the first step and BBE in the second.

Substrate	Procedure[a]	Time[b] [h]	(*S*)-2[c] [%]	*ee* (2)[d] [%]
**1 a**	A	48+24	93	>97
**1 a**	B	48+24	90	>97
**1 b**	A	48+24	97	>97
**1 b**	B	48+24	95	>97
**1 c**	A	72+24	79[e]	>97
**1 c**	B	72+24	79[e]	>97

[a] Procedure A: direct addition of BBE to the reaction mixture after completion of the first step. Procedure B: addition of BBE after removal of MAO by centrifugation.

[b] Reaction time for 1st+2nd step.

[c] Determined by HPLC on an achiral stationary phase.

[d] Determined by HPLC on a chiral stationary phase.

[e] 4 % of regioisomer formed.

Consequently, the two transformations were performed simultaneously in one pot. Thus, the two enantioselective oxidation reactions catalyzed by two different flavin-dependent enzymes (MAO and BBE) and the non-stereoselective morpholine–borane reduction were started concurrently for this cascade. It is noteworthy that in contrast to our previous studies on BBE,[7b] where catalase was required to protect the enzyme from oxidative inactivation by hydrogen peroxide, the cascade system did not profit from the addition of catalase.[15]

As the initial experiments indicated that the simultaneous reaction worked well for substrate *rac*-**1 a** and *rac*-**1 b**,[16] the synthetic applicability of the simultaneous cascade system was investigated in preparative-scale reactions (150–165 mg, 0.5 mmol). Excellent conversions were achieved for both substrates; for example, the deracemization of *rac*-**1 b** reached near-completion within 24 h (Table 2). The reaction products (*S*)-**2 a** and (*S*)-**2 b** were isolated in 88 % and 80 % yield, respectively, in optically pure form (*ee*>97 %).

**Table 2:** Deracemization of benzylisoquinolines *rac*-1 to berbines (*S*)-2­ *via* a cascade transformation using MAO, BBE, and morpholine-borane concurrently.[a]

Substrate	Time [h]	Conv. (2)[b] [%]	Yield (*S*)-2[c] [%]	*ee* (2)[d] [%]
**1 a**	48	92	88	>97
**1 b**	24	98	80	>97

[a] Reactions were performed in buffer/DMSO=90/10, pH 7.7; 10 mm **1**, 100 g L^−1^ *E. coli*/MAO-N D11, 0.05 g L^−1^ (**1 a**) or 0.02 g L^−1^ (**1 b**) BBE, 100 mm morpholine–borane, 37 °C.

[b] Determined by HPLC on an achiral stationary phase.

[c] Yield of islated product after column chromatography.

[d] Determined by HPLC on a chiral stationary phase.

In summary, a new concept to transform a racemic substrate to an optically pure product was established, whereby the substrate and product differ in structure. The concept combines stereoinversion of one substrate enantiomer with a kinetic resolution. In the example presented herein, the cascade comprises an enantioselective biocatalytic amine oxidation, a chemical non-stereoselective iminium ion reduction, and an enantioselective aerobic C=C bond formation to transform benzylisoquinolines *rac-***1** to berbines (*S*)-**2**. The three-step system led to conversions of up to 98 % with up to 88 % yield of isolated optically pure product (*S*)-**2** (*ee*>97 %, HPLC). Intriguingly, the two flavin-dependent oxidases employed act on different parts of the benzylisoquinoline substrate whereby each enzyme (MAO-N, BBE) preferentially transform the opposite enantiomer. The deracemization cascade was successfully demonstrated on preparative scale (150 mg), stimulating further developments for cascade reactions involving chemical and biocatalytic steps to avoid the disadvantages of kinetic resolution, thereby increasing the yield and making synthetic routes more economic and environmentally benign.

## Experimental Section

Representative MAO-N/BBE cascade transformation on preparative scale: In an Erlenmeyer flask (250 mL), lyophilized cells of *E. coli* C43(DE3) expressing MAO-N D11 (5.0 g) were resuspended in phosphate buffer (45 mL; 100 mm K-phosphate, pH 7.7). Morpholine⋅BH_3_ (505 mg, 5 mmol), BBE solution (105 μL of a 354 μm preparation; final conc. 0.05 g L^−1^) and a solution of substrate **1 e** (150 mg, 0.5 mmol; final conc. 10 mm) in DMSO (5 mL) were added, and the mixture was shaken at 37 °C and 150 rpm. After 24 h, additional morpholine⋅BH_3_ (250 mg, 2.5 mmol) and BBE solution (105 μL) were added, and shaking was continued. After 48 h, a sample (250 μL) was taken, extracted with EtOAc and analyzed for conversion. HPLC analysis showed 92 % product formation, and the reaction mixture was centrifuged (4000 rpm, 1 h, 21 °C) to remove the cell mass. The supernatant was extracted with EtOAc (3×20 mL), whereby phase separation was accelerated by centrifugation; the cell pellets were suspended in EtOAc, centrifuged again (4000 rpm, 10 min, 21 °C), and the EtOAc phase was combined with the extracts. The combined organic phases were dried over Na_2_SO_4_ and the solvent was evaporated under reduced pressure to give 0.60 g of a yellowish liquid. Column chromatography (silica gel, CH_2_Cl_2_/MeOH/NH_3_(aq)=96:3:1) afforded 131 mg (88 %) of (*S*)-**2 e** as a white solid (for product characterization, see the Supporting Information).

## References

[1] For reviews, see:

[1a] Hoyos P, Pace V, Alcántara AR (2012). Adv. Synth. Catal.

[1b] Pellissier H (2011). Tetrahedron.

[1c] Lee JH, Han K, Kim M-J, Park J (2010). Eur. J. Org. Chem.

[1d] Turner NJ (2010). Curr. Opin. Chem. Biol.

[1e] Kamaruddin AH, Uzir MH, Aboul-Enein HY, Halim HNA (2009). Chirality.

[1f] Ahn Y, Ko S-B, Kim M-J, Park J (2008). Coord. Chem. Rev.

[1g] Pellissier H (2008). Tetrahedron.

[1h] Turner NJ (2004). Curr. Opin. Chem. Biol.

[1i] Pàmies O, Bäckvall J-E (2003). Chem. Rev.

[2] Steinreiber J, Faber K, Griengl H, For a recent review, see: (2008). Chem. Eur. J.

[3] Selected examples:

[3a] Bailey KR, Ellis AJ, Reiss R, Snape T, Turner NJ (2007). Chem. Commun.

[3b] Dunsmore CJ, Carr R, Fleming T, Turner NJ (2006). J. Am. Chem. Soc.

[3c] Carr R, Alexeeva M, Enright A, Eve TSC, Dawson MJ, Turner NJ Angew. Chem.

[4] For reviews see:

[4a] Schrittwieser JH, Sattler J, Resch V, Mutti FG, Kroutil W (2011). Curr. Opin. Chem. Biol.

[4b] Gruber CC, Lavandera I, Faber K, Kroutil W (2006). Adv. Synth. Catal.

[5] For a recent review, see:

[5a] Schober M, Faber K (2013). Trends Biotechnol.

[6] For a recent example of a chemical enantioconvergent process, see:

[6a] Ito H, Kunii S, Sawamura M (2010). Nat. Chem.

[6b] Szymański W, Westerbeek A, Janssen DB, Feringa BL Angew. Chem.

[6c] Schober M, Gadler P, Knaus T, Kayer H, Birner-Grünberger R, Gülly C, Macheroux P, Wagner U, Faber K (2011). Org. Lett.

[6d] Schober M, Toesch M, Knaus T, Strohmeier GA, van Loo B, Fuchs M, Hollfelder F, Macheroux P, Faber K (2013). Angew. Chem.

[7a] Schrittwieser JH, Resch V, Sattler JH, Lienhart W-D, Durchschein K, Winkler A, Gruber K, Macheroux P, Kroutil W (2011). Angew. Chem.

[7b] Resch V, Schrittwieser JH, Wallner S, Macheroux P, Kroutil W (2011). Adv. Synth. Catal.

[7c] Schrittwieser JH, Resch V, Wallner S, Lienhart W-D, Sattler JH, Resch J, Macheroux P, Kroutil W (2011). J. Org. Chem.

[7d] Resch V, Lechner H, Schrittwieser JH, Wallner S, Gruber K, Macheroux P, Kroutil W (2012). Chem. Eur. J.

[7e] Schrittwieser JH, Resch V (2013). RSC Adv.

[8] For examples using ruthenium complexes, see:

[8a] Thalén LK, Hedberg MH, Bäckvall J-E (2010). Tetrahedron Lett.

[8b] Thalén LK, Zhao D, Sortais J-B, Paetzold J, Hoben C, Bäckvall J-E (2009). Chem. Eur. J.

[8c] Hoben CE, Kanupp L, Bäckvall J-E (2008). Tetrahedron Lett.

[8d] Veld MAJ, Hult K, Palmans ARA, Meijer EW (2007). Eur. J. Org. Chem.

[8e] Roengpithya C, Patterson DA, Livingston AG, Taylor PC, Irwin JL, Parrett MR (2007). Chem. Commun.

[8f] Paetzold J, Bäckvall JE (2005). J. Am. Chem. Soc.

[8g] Pàmies O, Éll AH, Samec JSM, Hermanns N, Bäckvall J-E (2002). Tetrahedron Lett.

[8h] Jerphagnon T, Gayet AJA, Berthiol F, Ritleng V, Mršić N, Meetsma A, Pfeffer M, Minnaard AJ, Feringa BL, de Vries JG (2009). Chem. Eur. J.

[8i] Blacker AJ, Stirling MJ, Page MI (2007). Org. Process Res. Dev.

[8j] Stirling M, Blacker J, Page MI (2007). Tetrahedron Lett.

[9] For examples using palladium on various supports, see:

[9a] Parvulescu AN, Jacobs PA, De Vos DE (2009). Appl. Catal. A.

[9b] Andrade LH, Silva AV, Pedrozo EC (2009). Tetrahedron Lett.

[9c] Parvulescu AN, Van der Eycken E, Jacobs PA, De Vos DE (2008). J. Catal.

[9d] Parvulescu AN, Jacobs PA, De Vos DE (2007). Chem. Eur. J.

[9e] Parvulescu A, De Vos D, Jacobs P (2005). Chem. Commun.

[9f] Reetz MT, Schimossek K (1996). Chimia.

[9g] Engström K, Johnston EV, Verho O, Gustafson KPJ, Shakeri M, Tai C-W, Bäckvall J-E Angew. Chem.

[9h] Geukens I, Plessers E, Seo JW, De Vos DE (2013). Eur. J. Inorg. Chem.

[9i] Shakeri M, Tai C-W, Göthelid E, Oscarsson S, Bäckvall J-E (2011). Chem. Eur. J.

[9j] Kim Y, Park J, Kim M-J (2010). Tetrahedron Lett.

[9k] Kim M-J, Kim W-H, Han K, Choi YK, Park J (2007). Org. Lett.

[9l] Parvulescu AN, Jacobs PA, De Vos DE (2008). Adv. Synth. Catal.

[10a] Poulhès F, Vanthuyne N, Bertrand MP, Gastaldi S, Gil G (2011). J. Org. Chem.

[10b] El Blidi L, Vanthuyne N, Siri D, Gastaldi S, Bertrand MP, Gil G (2010). Org. Biomol. Chem.

[10c] El Blidi L, Nechab M, Vanthuyne N, Gastaldi S, Bertrand MP, Gil G (2009). J. Org. Chem.

[10d] Routaboul L, Vanthuyne N, Gastaldi S, Gil G, Bertrand M (2008). J. Org. Chem.

[10e] Gastaldi S, Escoubet S, Vanthuyne N, Gil G, Bertrand MP (2007). Org. Lett.

[10f] Escoubet S, Gastaldi S, Vanthuyne N, Gil G, Siri D, Bertrand MP (2006). J. Org. Chem.

[10g] Escoubet S, Gastaldi S, Vanthuyne N, Gil G, Siri D, Bertrand MP (2006). Eur. J. Org. Chem.

[11] Warner MC, Casey CP, Bäckvall JE, For reviews, see: (2011). Top. Organomet. Chem.

[11b] Karvembu R, Prabhakaran R, Natarajan K (2005). Coord. Chem. Rev.

[11c] Shvo Y, Czarkie D, Rahamim Y, Chodosh DF (1986). J. Am. Chem. Soc.

[12] The transformation of (*S**R*

[12a] Hirata K, Poeaknapo C, Schmidt J, Zenk MH (2004). Phytochemistry.

[12b] De-Eknamkul W, Zenk MH (1992). Phytochemistry.

[12c] De-Eknamkul W, Zenk MH (1990). Tetrahedron Lett.

[13] Ghislieri D, Green AP, Pontini M, Willies SC, Rowles I, Frank A, Grogan G, Turner NJ (2013). J. Am. Chem. Soc.

[14] Steffens P, Nagakura N, Zenk MH (1984). Tetrahedron Lett.

[15] Apparently, the presence of the borane complex and the natural catalase activity of the *E. coli*_2__2_

[16] Substrate **1 c****2 c**

