# Supplementary material for: Deracemization By Simultaneous Bio-oxidative Kinetic Resolution and Stereoinversion
Source: Angew Chem Int Ed Engl. 2014 Feb 24;53(14):3731–4. doi: 10.1002/anie.201400027 (PMC4499246; doi:10.1002/anie.201400027)
Supplement: Supplementary file 1 [file anie0053-3731-sd1.pdf]

Angewandte Chemie
Eine Zeitschrift der Gesellschaft Deutscher Chemiker

Supporting Information
© Wiley-VCH 2014
69451 Weinheim, Germany

Deracemization By Simultaneous Bio-oxidative Kinetic Resolution and Stereoinversion**

Joerg H. Schrittwieser, Bas Groenendaal, Verena Resch, Diego Ghislieri, Silvia Wallner, Eva-Maria Fischereder, Elisabeth Fuchs, Barbara Grischek, Johann H. Sattler, Peter Macheroux, Nicholas J. Turner, and Wolfgang Kroutil**

anie_201400027_sm_miscellaneous_information.pdf

Supporting Information

Contents:

Supplementary Data S2
Oxidation of Benzylisoquinolines by MAO-N D11 .... S2
Effect of Amine-Borane Complexes on BBE Activity S2
Combination of MAO-N D11 with Varied Amine-Boranes S2
Supplementary Methods S4
General Methods S4
Synthesis of Substrates S4
Protein Expression and Purification S5
Substrate Screening of MAO-N Variants S6
Biotransformations S7
Analytical Methods S9
NMR and MS Spectra S11
Substrate **1f** and Its Synthetic Intermediates S12
Products of Enzymatic Conversions S29
References S40

Supplementary Data

Oxidation of Benzylisoquinolines by MAO-N D11

The enantioselectivity of MAO-N D11 towards benzylisoquinolines **1** was determined from the conversion and the *ee* of the remaining substrate. As shown in Supplementary Table S1, MAO-N D11 preferentially oxidized the (*R*)-enantiomer of compounds **1a–c**, while the enantioselectivity for **1d** was low.

substrate	conversion [%][a]	*ee* (1) [%][b]	*E*[c]
**1a**	28.7	40 (*S*)	> 200
**1b**	26.0	35 (*S*)	> 200
**1c**	15.2	18 (*S*)	> 200
**1d**	26.2	24 (*S*)	6.5

Supplementary Table S1. Results of MAO-catalyzed oxidation reactions. [a] Determined by HPLC analysis using an internal standard. [b] Determined by HPLC analysis on a chiral stationary phase. [c] Determined form conversion (c) and substrate *ee* (ee_S) according to the equation: $E = \ln[(1-c)\cdot(1-ee_S)]/\ln[(1-c)\cdot(1+ee_S)]$.[1]

Effect of Amine-Borane Complexes on BBE Activity

The conversion of *rac*-1-(3-hydroxybenzyl)-6,7-dimethoxy-2-methyl-1,2,3,4-tetrahydroisoquinoline (**1e**) by BBE was carried out in the presence of varied amounts of amine-borane complexes to evaluate their effect on BBE activity (Supplementary Figure S1).

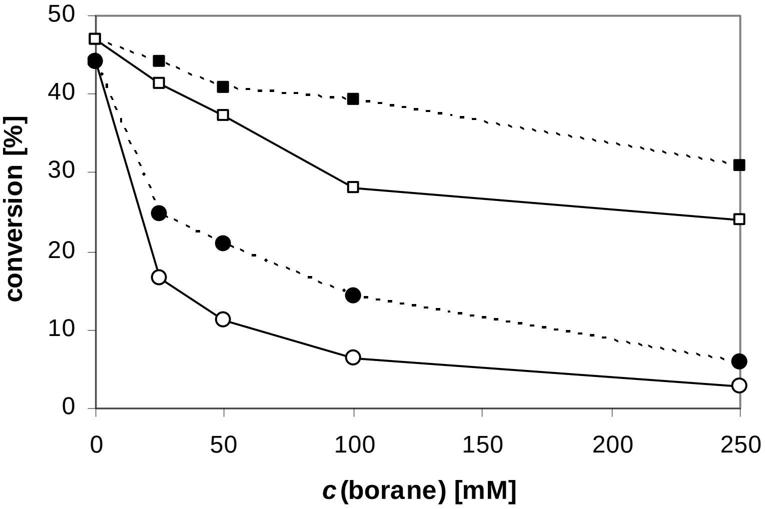

Supplementary Figure S1. Conversion of *rac*-**1e** to (*S*)-**2e** by BBE in the presence of varied amounts of amine-borane complexes $NH_3\cdot BH_3$ (–○–), $^tBuNH_2\cdot BH_3$ (--●--), morpholine·BH_3 (–□–), or $Me_3N\cdot BH_3$ (--■--). Reaction time: 2 h.

Combination of MAO-N D11 with Various Amine-Boranes

The effect of the borane source on the efficiency of MAO-N-catalyzed stereoinversion reactions was studied using *rac*-1-benzyl-6,7-dimethoxy-2-methyl-1,2,3,4-tetrahydroisoquinoline (**1f**) as substrate, since its oxidation product[2] can be extracted and quantified by GC–FID analysis. Hence, incomplete reduction of the MAO-generated prochiral intermediate can be easily detected.

As shown in Supplementary Figure S2, $NH_3{\cdot}BH_3$ and ${}^tBuNH_2{\cdot}BH_3$ performed very similar in the stereoinversion system, while the trimethylamine and the morpholine complex both gave superior results. In the reactions with $Me_3N{\cdot}BH_3$, however, incomplete reduction was observed when the borane source was applied in concentrations lower than 250 mM. Hence, we chose to use morpholine-borane in all further experiments.

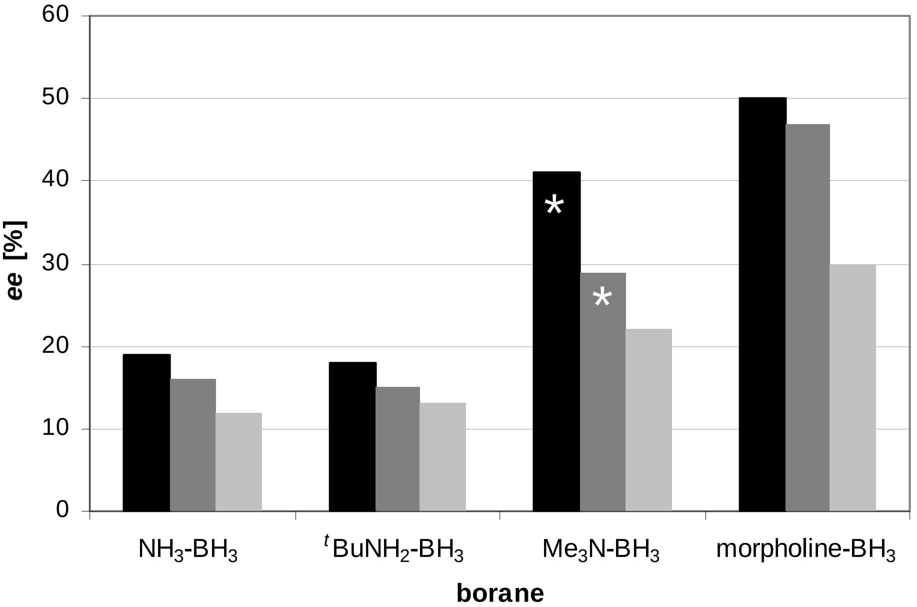

Supplementary Figure S2. Stereoinversion of *rac*-**1f** using MAO-N D11 in the presence of varied amounts of amine-borane complexes: 50 mM (■), 100 mM (■), or 250 mM (■). The asterisk indicates samples where incomplete reduction was observed (50 mM $Me_3N{\cdot}BH_3$: 5.1% oxidation product, 100 mM $Me_3N{\cdot}BH_3$: 2.8% oxidation product). Reaction time: 20 h.

Supplementary Methods

General Methods

^{1}H- and ^{13}C-NMR spectra were recorded using a 300 MHz instrument. Chemical shifts are given in parts per million (ppm) relative to TMS (δ = 0 ppm) and coupling constants (J) are reported in Hertz (Hz). Melting points were determined on a Gallenkamp MPD350 apparatus in open capillary tubes and are uncorrected. Thin layer chromatography was carried out on silica gel 60 F_{254} plates and compounds were visualized either by UV. Unit resolution GC–MS analyses were performed using electron impact (EI) ionisation at 70 eV and quadrupole mass selection. High resolution MS analyses were performed using electron impact (EI) ionisation at 70 eV and TOF mass selection. Optical rotation values $[\alpha]_D^{20}$ were measured at 589 nm (Na line) on a Perkin-Elmer Polarimeter 341 using a cuvette of 1 dm path length, and are given in ° L g^{-1} m^{-1}.

Unless otherwise noted, reagents and organic solvents were purchased from commercial suppliers in reagent grade quality, and were used without further purification.

Synthesis of Substrates

Benzylisoquinolines *rac*-**1a–e** and berbines (*S*)-**2c**, and (*S*)-**2d** were synthesized as previously described.[3] Benzylisoquinoline *rac*-**1f** was prepared as described below:

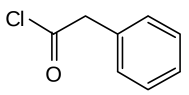

Phenylacetyl chloride. A solution of phenylacetic acid (2.00 g, 14.7 mmol), oxalyl chloride (2.81 g, 22.1 mmol) and one drop of DMF in dry toluene (40 mL) was stirred at room temperature under argon for 1 h. The solvent was evaporated under reduced pressure to give 2.27 g (quant.) of phenylacetyl chloride as a brownish liquid, which was used in the following transformation without further purification. ^{1}H-NMR ($CDCl_3$, 300 MHz): δ = 4.17 (2H, s, CH_2-COCl), 7.28–7.43 (5H, m, Ar). ^{13}C-NMR ($CDCl_3$, 75 MHz): δ = 53.1, 128.2, 129.0, 129.4, 129.5, 131.2, 171.9. The NMR data are in accordance with literature.[4]

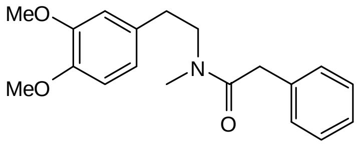

***N*-(3,4-Dimethoxyphenethyl)-*N*-methyl-2-phenylacetamide.** *N*-Methylhomoveratrylamine (3.11 g, 15.9 mmol) was dissolved in $CHCl_3$ (30 mL). 3% aq. NaOH solution (150 mL) was added and the mixture was cooled to 0 °C on an ice bath. A solution of phenylacetyl chloride (2.25 g, 14.6 mmol) in chloroform (30 mL) was added dropwise over 1 h to the vigorously stirred mixture. The ice bath was then removed and stirring was continued overnight at room temperature. The phases were separated and the aqueous phase was extracted with $CHCl_3$ (50 mL). The combined organic phases were washed with 1 M aq. HCl solution (100 mL), then water (100 mL), and dried over Na_2SO_4. Evaporation of the solvent under reduced pressure yielded 4.67 g of a highly viscous yellow liquid. Flash chromatography (silica gel 60; petrol ether/EtOAc = 1/1) afforded *N*-(3,4-dimethoxyphenethyl)-*N*-methyl-2-phenylacetamide (3.93 g, 85%) as a yellowish liquid. TLC (petrol ether/EtOAc = 1/1): R_f = 0.25. MS (EI, 70 eV): m/z = 313 (M^+, 3), 164 (100), 151 (12), 91 (30). HRMS calcd for $C_{19}H_{23}NO_3$: 313.1678; found: 313.1703.

NMR spectroscopy reveals that the product is a mixture of isomers (ratio *trans*/*cis* = 1.25/1). Based on the peak intensities as well as the DEPT, COSY and HMQC spectra, the NMR signals can be assigned to the isomers as follows:

trans-*N*-(3,4-Dimethoxyphenethyl)-*N*-methyl-2-phenylacetamide. ^{1}H-NMR ($CDCl_3$, 300 MHz): δ = 2.71 (2H, t, J = 7.5 Hz, CH_2-CH_2-N), 2.79 (3H, s, N-CH_3), 3.51 (2H, t, J = 7.5 Hz,

CH_2-CH_2-N), 3.60 (2H, s, CH_2-CO), 3.73 (3H, s, OCH_3), 3.77 (3H, s, OCH_3), 6.60–6.69 (3H, m, Ar), 7.06–7.25 (5H, m, Ar). ^{13}C-NMR ($CDCl_3$, 75 MHz): δ = 33.2 (CH_2), 36.5 (CH_3), 41.3 (CH_2), 50.1 (CH_2), 55.8 (CH_3), 55.9 (CH_3), 111.3 (CH), 112.0 (CH), 120.7 (CH), 126.7 (CH), 128.6 (CH), 128.7 (CH), 131.6 (C), 135.0 (C), 147.5 (C), 148.9 (C), 170.8 (C). No NMR reference data were available for this compound.

cis-*N*-(3,4-Dimethoxyphenethyl)-*N*-methyl-2-phenylacetamide. ^{1}H-NMR ($CDCl_3$, 300 MHz): δ = 2.57 (2H, t, J = 7.2 Hz, CH_2-CH_2-N), 2.90 (3H, s, N-CH_3), 3.37–3.42 (4H, t + s overlap, CH_2-CH_2-N + CH_2-CO), 3.76 (3H, s, OCH_3), 3.77 (3H, s, OCH_3), 6.48 (1H, d, J = 2.0 Hz, Ar-2), 6.54 (1H, dd, J_1 = 8.1 Hz, J_2 = 2.0 Hz, Ar-6), 6.72 (1H, d, J = 8.1 Hz, Ar-5), 7.06–7.25 (5H, m, Ar). ^{13}C-NMR ($CDCl_3$, 75 MHz): δ = 33.6 (CH_3), 34.3 (CH_2), 40.7 (CH_2), 52.2 (CH_2), 55.8 (CH_3), 55.9 (CH_3), 111.5 (CH), 112.0 (CH), 120.8 (CH), 126.7 (CH), 128.6 (CH), 128.7 (CH), 130.7 (C), 135.3 (C), 147.9 (C), 149.1 (C), 171.0 (C). No NMR reference data were available for this compound.

1-Benzyl-6,7-dimethoxy-2-methyl-1,2,3,4-tetrahydroisoquinoline (1f). A solution of *N*-(3,4-dimethoxyphenethyl)-*N*-methyl-2-phenylacetamide (3.93 g, 12.6 mmol) and $POCl_3$ (5.88 g, 38.4 mmol) in dry acetonitrile (50 mL) was refluxed for 4 h under argon atmosphere. The solvent and excess $POCl_3$ were evaporated under reduced pressure, and the residue was dissolved in dry methanol (50 mL), put under argon, and cooled to 0 °C on an ice bath. $NaBH_4$ (2.38 g, 62.9 mmol) was added in portions to the stirred mixture. The ice bath was then removed and stirring continued for 16 h at room temperature (21 °C). The solvent was evaporated, and the residue was treated with half-saturated aq. Na_2CO_3 solution (100 mL). The product was extracted with CH_2Cl_2 (3 × 30 mL), the combined organic phases were dried over Na_2SO_4 and evaporated under reduced pressure to give 3.71 g of a yellow liquid. Flash chromatography (silica gel 60; CH_2Cl_2/MeOH/NH_3(aq) = 95/4/1) afforded 1-benzyl-6,7-dimethoxy-2-methyl-1,2,3,4-tetrahydroisoquinoline (3.34 g, 89%) as a yellowish liquid that crystallized upon standing in the fridge to an off-white solid. mp: 70–71 °C (ref.[5] 79–81 °C). TLC (CH_2Cl_2/MeOH/NH_3(aq) = 90/9/1): R_f = 0.59. ^{1}H-NMR ($CDCl_3$, 300 MHz): δ = 2.57 (3H, s, NCH_3), 2.61–2.68 (1H, m CH_2), 2.78–2.88 (3H, m, CH_2), 3.23–3.31 (2H, m, CH_2), 3.51 (3H, s, OCH_3), 3.74–3.79 (1H, m, CH), 3.85 (3H, s, OCH_3), 5.94 (1H, s, Ar), 6.58 (1H, s, Ar), 7.11–7.30 (5H, m, Ar). ^{13}C-NMR ($CDCl_3$, 75 MHz): δ = 25.3, 41.2, 42.5, 46.6, 55.4, 55.7, 64.9, 111.0, 111.1, 125.5, 126.0, 128.2, 128.9, 129.9, 129.9, 146.2, 147.3. MS (EI, 70 eV): m/z = 296 (M^+–H, <1), 206 (100), 190 (15), 162 (5). HRMS calcd for $C_{19}H_{22}NO_2$ (M^+–H): 296.1650; found: 296.1683. The NMR data are in accordance with literature.[6]

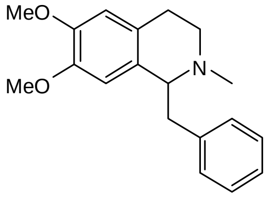

Protein Expression and Purification

Expression and purification of berberine bridge enzyme (BBE) were carried out as previously described.[3b,7] Expression and purification of MAO-N D11 were performed as described below:

Cultivation of *E. coli* strains expressing MAO-N (for protein purification). A single colony of *E. coli* BL21(DE3) harboring the [pET16b MAO-N (D11)] plasmid was used to inoculate 8 mL of LB medium (containing 100 µg/mL ampicillin) and the culture was grown to an OD_{600} of 0.6–1.0 at 30 °C and 250 rpm. From this culture, 6 mL were used to inoculate 600 mL of LB medium (containing 100 µg/mL ampicillin) and the new culture was grown at 30 °C and 250 rpm for 24 h. The cells were harvested by centrifugation (5500 rpm, 20 min), resuspended in phosphate buffer (100 mM K-P_i, pH 7.7) and centrifuged again (4000 rpm, 15 min). The cell pellets were stored at –20 °C until use.

Purification of MAO-N. Protein purification was performed on an ÄKTAexplorer 900 system (*GE Healthcare*) at 10 °C according to the following protocol: 5 g of cell pellet prepared as described above were resuspended in 25 mL of phosphate buffer A (100 mM K-P_i, pH 7.7, 300 mM NaCl) containing 1 mg/mL of lysozyme and incubated at 30 °C for 30 min. The suspension was cooled to 4 °C and cells were disrupted by ultrasonication (*MSE* Soniprep 150; 30 s pulse, 30 s pause; 15 cycles). The cell debris was removed by centrifugation (20000 rpm, 40 min), the supernatant was filtered (0.45 µm syringe microfilter) and loaded onto a HisTrap Ni-sepharose column (1 mL, *GE Healthcare*) pre-equilibrated with buffer A (100 mM K-P_i, pH 7.7, 300 mM NaCl). Proteins were step-eluted using buffer A (10 mL), buffer A / buffer B (100 mM K-P_i, pH 7.7, 300 mM NaCl, 1 M imidazole) = 80/20 (10 mL), and buffer A / buffer B = 65/35 (10–30 mL); collecting 1 mL fractions. The MAO-containing fractions (from the 35% buffer B step) were pooled and concentrated using the *Sartorius* Vivaspin 6 system (30 kDa mass cut-off), the volume was adjusted to 2.5 mL, and the protein solution was desalted using a PD-10 column (*GE Healthcare*) and MAO reaction buffer (100 mM K-P_i, pH 7.7) for elution. The protein solution thus prepared was directly used for the colorimetric substrate screening. Protein concentration was determined using the Pierce BCA protein assay (*Thermo Scientific*) preparing all samples (MAO and BSA standard) in triplicate.

Cultivation under auto-inducing conditions. LB medium (50 mL; containing 100 µg/mL ampicillin) was inoculated from a glycerol stock (20 µL) of *E. coli* C43(DE3) harboring the [pET16b MAO-N (D11)] plasmid, and the culture was grown to an OD_{600} >2 at 30 °C and 120 rpm (approx. 16 h). From this culture, 6 mL were used to inoculate autoinduction medium (600 mL, 4ZY-LAC-SUC; containing 100 µg/mL ampicillin), and the new culture was grown at 25 °C and 150 rpm for 72 h. The cells were harvested by centrifugation (8000 rpm, 20 min), resuspended in phosphate buffer (100 mM K-P_i, pH 7.7), and centrifuged again (4000 rpm, 20 min). The cell pellets were stored at –20 °C.

Cell lyophilization. Cell pellets (approx. 2 g) prepared as described above were resuspended in 10 mL of phosphate buffer (100 mM K-P_i, pH 7.7). The cell suspension was transferred to a round-bottom flask and flash-frozen by submerging the rotating flask into a bath of liquid nitrogen. The resulting frozen cell suspension was lyophilized over night. The lyophilisate thus prepared was stored at 4 °C and used for oxidation and deracemisation reactions.

Media and media components:

LB medium: 10 g/L tryptone, 5 g/L yeast extract, 10 g/L NaCl

4ZY-LAC-SUC: 20 mL/L 50x LAC, 50 mL/L 20x NPSC, 1 mL/L 1000x trace element solution, 2 mL/L 500x $MgSO_4$ stock, 50 mL/L 20x SUC stock, 410 mL/L 8x ZY, 470 mL/L H_2O dest.

50x LAC: 25% w/v glycerol, 2.5% w/v glucose, 10% w/v α-lactose monohydrate

20x NPSC: 1 M NH_4Cl, 0.1 M Na_2SO_4, 0.5 M KH_2PO_4, 0.5 M Na_2HPO_4

1000x trace element solution: 50 mM $FeCl_3$, 20 mM $CaCl_2$, 10 mM $MnCl_2$, 10 mM $ZnSO_4$, 2 mM $CoCl_2$, 2 mM $CuCl_2$, 2 mM $NiCl_2$, 2 mM Na_2MoO_4, 2 mM Na_2SeO_3, 2 mM H_3BO_4

500x $MgSO_4$ stock: 1 M $MgSO_4$

20x SUC: 0.5 M sodium succinate

8x ZY: 80 g/L tryptone, 40 g/L yeast extract

Substrate Screening of MAO-N D11

Stock solutions of the test substrates (20 mM; final concentration after dilution 1 mM) in DMSO were prepared. For each colorimetric reaction, the substrate stock solution (5 µL) was mixed with MAO reaction buffer (20 µL; 100 mM K-P*i*, pH 7.7), pyrogallol red solution (50 µL; 0.3 mM in MAO reaction buffer), horseradish peroxidase solution (5 µL; 1 mg/mL in MAO reaction buffer) and purified MAO-N D11 solution (20 µL; 0.53 mg/mL) in a 96-well microtiter plate. In addition, reactions with *rac*-1-phenylethylamine (1 mM) and *rac*-crispine A (1 mM), as well as negative controls (lacking substrate and MAO, respectively) were set up. All reactions, including the blanks, were performed in triplicate. Reactions were followed by measuring the absorbance at 550 nm every minute for a period of 4 h using a *Molecular Devices* SpectraMax M2 plate reader. Slopes were determined by applying a linear fit to the linear range of the absorbance curve using the built-in function of the plate reader's *Molecular Devices* Softmax Pro v5.0 software. Slopes were corrected for spontaneous decolorization of pyrogallol red (rate obtained from the blank samples) and the specific MAO activity was calculated using formula (1) given below.

$$A = \frac{\Delta OD \cdot Y}{\varepsilon \cdot L \cdot c_{\mathrm{P}}} \qquad (1)$$

where A [U·mg^{-1}] … MAO activity; ΔOD [min^{-1}] … slope of absorbance decrease; Y … dilution factor of MAO solution (5 in this case); ε [L·mmol^{-1}·cm^{-1}]… extinction coefficient of pyrogallol red (30.9 at 550 nm); L [cm] … path length of sample (0.31 in this case); c_{P} [mg·mL^{-1}] … protein concentration of MAO stock solution.

Biotransformations

Investigation of the effect of different amine-borane complexes on BBE activity. In Eppendorf tubes (2 mL), the appropriate amount of amine-borane ($NH_3 \cdot BH_3$, $^t BuNH_2 \cdot BH_3$, morpholine·BH_3, or $Me_3N \cdot BH_3$; 12.5, 25, 50, or 125 µmol; final concentration: 25, 50, 100, or 250 mM) was dissolved in reaction buffer (450 µL; 100 mM K-phosphate, pH 7.7). BBE solution (2.1 µL of a 354 µM preparation; final concentration: 0.1 g/L) and a solution of *rac*-1-(3-hydroxybenzyl)-6,7-dimethoxy-2-methyl-1,2,3,4-tetrahydroisoquinoline (**1e**; 1 mg, 3.2 µmol; final concentration: 6.4 mM) in DMSO (50 µL) were added and the mixture was shaken at 37 °C and 150 rpm. In a similar manner, a blank sample without any amine-borane was prepared. After 2 h, the samples were extracted with EtOAc (2 × 500 µL) and the extract was dried over Na_2SO_4. The solvent was evaporated under a stream of air, the samples were re-dissolved in HPLC-grade methanol (700 µL) and conversion was determined by HPLC analysis.

Investigation of the effect of different amine-borane complexes on the efficiency of MAO-catalyzed deracemisation. In Eppendorf tubes (2 mL), lyophilized cells of *E. coli* C43(DE3) expressing MAO-N D11 (15 mg) were resuspended in phosphate buffer (450 µL; 100 mM K-P$_i$, pH 7.7). The appropriate amount of amine-borane ($NH_3 \cdot BH_3$, $^t BuNH_2 \cdot BH_3$, morpholine·BH_3, or $Me_3N \cdot BH_3$; 25, 50, or 125 µmol; final concentration: 50, 100, or 250 mM) and a solution of *rac*-1-benzyl-6,7-dimethoxy-2-methyl-1,2,3,4-tetrahydroisoquinoline (**1f**; 1.5 mg, 5 µmol; final concentration: 10 mM) in DMSO (50 µL) were added and the mixture was shaken at 37 °C and 150 rpm. After 20 h, the sample was extracted with EtOAc (2 × 500 µL) and the extract was dried over Na_2SO_4. The solvent was evaporated under a stream of air and the sample was re-dissolved in HPLC-grade methanol (700 µL). Substrate *ee* was determined by HPLC analysis, and GC-FID analysis was used to analyze for presence of the oxidation product.

Stepwise combination of MAO-catalyzed deracemization and BBE-catalyzed cyclization.
Procedure A: Direct addition of BBE to the reaction mixture after complete deracemization: In Falcon tubes (15 mL), lyophilized cells of *E. coli* C43(DE3) expressing MAO-N (D11) (250 mg) were resuspended in reaction buffer (2.25 mL; 100 mM K-P_i, pH 7.7). Morpholine·BH_3 (25.3 mg, 250 µmol; final concentration 100 mM) and a solution of substrate **1** (25 µmol; final concentration 10 mM) in DMSO (250 µL) were added to the cell suspension. The reaction mixture was shaken at 37 °C and 150 rpm. Additional morpholine·BH_3 (15 mg, 150 µmol) was added after 24 and 48 h. After 48 h (**1a**, **1b**) or 72 h (**1c**), an aliquot (500 µL) was taken and BBE solution (2.1 µL of a 354 µM preparation; final concentration: 0.1 g/L) was added. The reaction was allowed to proceed for further 24 h at 37 °C and 150 rpm. Afterwards, the sample was extracted with EtOAc (2 × 500 µL) and the extract dried over Na_2SO_4. The solvent was evaporated under a stream of air, the sample was re-dissolved in HPLC-grade methanol (700 µL) and conversion as well as product *ee* were determined by HPLC analysis.
Procedure B: Addition of BBE to the supernatant of centrifuged reaction mixture after complete deracemization: In Falcon tubes (15 mL), lyophilized cells of *E. coli* C43(DE3) expressing MAO-N (D11) (250 mg) were resuspended in reaction buffer (2.25 mL; 100 mM K-P_i, pH 7.7). Morpholine·BH_3 (25.3 mg, 250 µmol; final concentration 100 mM) and a solution of substrate **1** (25 µmol; final concentration 10 mM) in DMSO (250 µL) were added to the cell suspension. The reaction mixture was shaken at 37 °C and 150 rpm. Additional morpholine·BH_3 (15 mg, 150 µmol) was added after 24 and 48 h. After 72 48 h (**1a**, **1b**) or 72 h (**1c**), an aliquot (1 mL) was taken and centrifuged (13,000 rpm, 2 min). From the supernatant, 500 µL were transferred to a fresh Eppendorf vial (2 mL) and BBE solution (2.1 µL of a 354 µM preparation; final concentration: 0.1 g/L) was added. The reaction was allowed to proceed for further 24 h at 37 °C and 150 rpm. Afterwards, the sample was extracted with EtOAc (2 × 500 µL) and the extract dried over Na_2SO_4. The solvent was evaporated under a stream of air, the sample was re-dissolved in HPLC-grade methanol (700 µL) and conversion as well as product *ee* were determined by HPLC analysis.

MAO/BBE cascade transformation of *rac*-1a (preparative scale). In an Erlenmeyer flask (250 mL), lyophilized cells of *E. coli* C43(DE3) expressing MAO-N D11 (5.0 g) were resuspended in phosphate buffer (45 mL; 100 mM K-phosphate, pH 7.7). Morpholine·BH_3 (505 mg, 5 mmol), BBE solution (105 µL of a 354 µM preparation; final concentration: 0.05 g/L) and a solution of substrate **1a** (150 mg, 0.5 mmol; final concentration 10 mM) in DMSO (5 mL) were added and the mixture was shaken at 37 °C and 150 rpm. After 24 h, additional morpholine·BH_3 (250 mg, 2.5 mmol) and BBE solution (105 µL) were added and shaking continued. After 48 h, a sample (250 µL) was taken, extracted with EtOAc and analyzed for conversion. HPLC analysis showed 92% product formation, and the reaction mixture was aliquoted into Falcon tubes (50 mL) and centrifuged (4000 rpm, 1 h, rt) to remove the cells. The supernatant was extracted with EtOAc (3 × 20 mL), whereby phase separation was accelerated by centrifugation; the cell pellets were suspended in EtOAc, centrifuged again (4000 rpm, 10 min, rt) and the EtOAc phase was combined with the extracts. The combined organic phases were dried over Na_2SO_4 and the solvent was evaporated under reduced pressure to give 0.60 g of a yellowish liquid. Column chromatography (silica; CH_2Cl_2/MeOH/NH_3(aq) = 97/2/1) afforded 131 mg (88%) of (*S*)-**2a** as a white solid foam. mp: 136 °C (decomp.). TLC (CH_2Cl_2/MeOH/NH_3(aq) = 90/9/1): $R_f = 0.62$. $[\alpha]_D^{20} = -281$ (c = 0.275, $CHCl_3$). ^{1}H-NMR (DMSO-d$_6$, 300 MHz): δ = 2.41–2.63 (3H, m, CH_2), 2.86–2.97 (1H, m, CH_2), 3.05–3.09 (1H, m, CH_2), 3.16–3.27 (2H, dd + d overlap, CH_2), 3.34–3.39 (1H, m, CH), 3.74 (3H, s, OCH_3), 3.98 (1H, d, J = 15.7 Hz, CH_2), 6.59–6.64 (3H, m, Ar), 6.71 (1H, s, Ar), 6.95 (1H, t, J = 7.7 Hz, Ar), 8.70 (1H, s, OH), 9.39 (1H, s, OH). ^{13}C-NMR (DMSO-d$_6$, 75 MHz): δ = 29.0, 37.0, 51.7, 54.0, 56.0, 58.9, 112.1, 112.2, 112.9, 119.5, 122.1, 125.2,

126.7, 130.4, 136.3, 145.0, 146.5, 153.7. MS (EI, 70 eV): m/z = 297 (100) [M^+], 296 (96) [M^+–H], 282 (15), 178 (61), 176 (75), 163 (17), 120 (19), 91 (19).

MAO/BBE cascade transformation of *rac*-1b (preparative scale). In an Erlenmeyer flask (250 mL), lyophilized cells of *E. coli* C43(DE3) expressing MAO-N D11 (5.0 g) were resuspended in phosphate buffer (45 mL; 100 mM K-phosphate, pH 7.7). Morpholine·BH_3 (505 mg, 5 mmol), BBE solution (42 µL of a 354 µM preparation; final concentration: 0.02 g/L) and a solution of substrate **1b** (165 mg, 0.5 mmol; final concentration 10 mM) in DMSO (5 mL) were added and the mixture was shaken at 37 °C and 150 rpm. After 24 h, a sample (250 µL) was taken, extracted with EtOAc and analyzed for conversion. HPLC analysis showed 98% product formation, and the reaction mixture was aliquoted into Falcon tubes (50 mL) and centrifuged (4000 rpm, 1 h, rt) to remove the cells. The supernatant was extracted with EtOAc (3 × 20 mL), whereby phase separation was accelerated by centrifugation; the cell pellets were suspended in EtOAc, centrifuged again (4000 rpm, 10 min, rt) and the EtOAc phase was combined with the extracts. The combined organic phases were dried over Na_2SO_4 and the solvent was evaporated under reduced pressure to give 0.54 g of a yellowish liquid. Column chromatography (silica; CH_2Cl_2/MeOH/NH_3(aq) = 97/2/1) afforded 131 mg (80%) of (*S*)-**2b** as a white solid foam. mp: 191–192 °C. TLC (CH_2Cl_2/MeOH/NH_3(aq) = 90/9/1): R_f = 0.55. $[\alpha]_D^{20}$ = −248 (c = 0.24, $CHCl_3$). ^{1}H-NMR (DMSO-d_6, 300 MHz): δ = 2.41–2.60 (3H, m, CH_2), 2.85–2.96 (1H, m, CH_2), 3.05–3.10 (1H, m, CH_2), 3.16 (1H, dd, J_1 = 15.7 Hz, J_2 = 3.5 Hz, CH_2), 3.25 (1H, d, J = 15.6 Hz, CH_2), 3.33 (1H, dd, J_1 = 11.5 Hz, J_2 = 3.3 Hz, CH), 3.73 (3H, s, OCH_3), 3.75 (3H, s, OCH_3), 4.01 (1H, d, J = 15.7 Hz, CH_2), 6. 58 (1H, d, J = 8.2 Hz, Ar), 6.63 (1H, s, Ar), 6.70 (1H, s, Ar), 6.77 (1H, d, J = 8.3 Hz, Ar), 8.59 (1H, s, OH), 8.70 (1H, s, OH). ^{13}C-NMR (DMSO-d_6, 75 MHz): δ = 29.0, 36.4, 51.7, 54.2, 56.0, 56.4, 59.1, 110.4, 112.2, 112.9, 119.0, 122.6, 125.2, 128.1, 130.4, 142.4, 145.1, 145.3, 146.4. MS (EI, 70 eV): m/z = 327 (74) [M^+], 326 (50) [M^+–H], 312 (9), 310 (8), 178 (100), 176 (29), 163 (10), 150 (41), 135 (19), 107 (11).

Analytical Methods

HPLC analysis (achiral stationary phase). HPLC analysis was carried out on a *Shimadzu* HPLC system using a C18 stationary phase [*Phenomenex* LUNA-C18(2), 250 mm × 4.6 mm] and diode array detection. Eluent: buffer (30 mM $HCOONH_4$, pH 2.8)/methanol/acetonitrile = 67/18/15; column temperature: 20 °C; flow rate: 0.5 mL/min; integration wavelength: 280 nm. The retention times of all investigated compounds are given in Supplementary Table S2:

substrate	t_r (1) [min]	t_r (2) [min]	t_r (RI) [min]
**1a**	8.6	9.4	–
**1b**	8.9	10.4	–
**1c**	18.8	23.1	12.6
**1d**	16.4	19.0	11.0
**1e**	13.0	15.9	9.9
**1f**	24.8	–	–

Supplementary Table S2. HPLC retention times of substrates **1**, main products **2**, and regioisomeric products **RI**.

HPLC analysis (chiral stationary phase). HPLC analysis of substrates **1** was carried out on a *Shimadzu* HPLC system using a [cellulose-tris(4-methyl-benzoate) on silica gel] stationary phase (*Daicel* Chiralcel OJ, 250 mm × 4.6 mm) and diode array detection.

Method A. Eluent: *n*-heptane/2-propanol = 70/30 (+0.1% formic acid); column temperature: 18 °C; flow rate: 0.35 mL/min; integration wavelength: 280 nm.

Method B. Eluent: *n*-heptane/2-propanol = 70/30 (+0.1% formic acid); column temperature: 18 °C; flow rate: 0.5 mL/min; integration wavelength: 280 nm.
Method C. Eluent: *n*-heptane/2-propanol = 80/20 (+0.1% TFA); column temperature: 40 °C; flow rate: 0.35 mL/min; integration wavelength: 280 nm.
The retention times of all investigated compounds are given in Supplementary Table S3:

substrate	method	t_r(*S*) [min]	t_r(*R*) [min]
**1a**	A	32.9	40.0
**1b**	B	28.6	32.6
**1c**	C	22.3	27.3

Supplementary Table S3. HPLC retention times of substrate **1** enantiomers.

HPLC analysis of products **2** was carried out on a *Shimadzu* HPLC system using an [amylose-tris(3,5-dimethylphenylcarbamate) on silica gel] stationary phase (*Daicel* Chiralpak AD, 250 mm × 4.6 mm) and diode array detection.
Method A. Eluent: *n*-heptane/2-propanol = 70/30; column temperature: 18 °C; flow rate: 0.5 mL/min; integration wavelength: 280 nm.
Method B. Eluent: *n*-heptane/2-propanol = 70/30; column temperature: 18 °C; flow rate: 0.75 mL/min; integration wavelength: 280 nm.
The retention times of all investigated compounds are given in Supplementary Table S4:

substrate	method	t_r(*R*) [min]	t_r(*S*) [min]
**2a**	A	16.0	23.8
**2b**	B	18.1	38.2
**2c**	A	10.2	15.9

Supplementary Table S4. HPLC retention times of product **2** enantiomers.

GC–FID analysis. GC–FID analysis was carried out on an *Agilent* 7890a GC system using a (5%-phenyl)methylpolysiloxane stationary phase (*J&W* HP-5, 30 m × 0.32 mm × 0.25 µm) and helium as carrier gas. *Method B.* Oven program: 200 °C for 0.5 min, 10 °C/min to 300 °C, keep for 4 min; column flow rate: 2 mL/min; split ratio: 15/1.
t_r (**1f**): 6.0 min; t_r (oxidation product of **1f**): 7.6 min.

Determination of absolute configuration. Absolute configurations of substrates **1** and products **2** were assigned based on optical rotation, circular dichroism and HPLC elution order analogies as previously described.[3,7b]

NMR and MS Spectra

NMR and MS spectra of chemically synthesized benzylisoquinolines *rac*-**1a–d** have been previously reported.[3] The spectra of compound *rac*-**1f** and its synthetic intermediates, as well as compounds (*S*)-**2a** and (*S*)-**2b** isolated from preparative scale MAO/BBE-cascade transformations are provided in this section of the Supporting Information.

Substrate 1f and its Synthetic Intermediates

Provided Material:

Phenylacetyl chloride:

^{1}H-NMR spectrum, ^{13}C-NMR spectrum

***N*-(3,4-Dimethoxyphenethyl)-*N*-methyl-2-phenylacetamide:**

^{1}H-NMR spectrum, ^{13}C-NMR spectrum, ^{13}C-NMR DEPT135 spectrum, ^{13}C-NMR DEPT90 spectrum, COSY spectrum, HSQC spectrum, MS spectrum, HRMS results

1-Benzyl-6,7-dimethoxy-2-methyl-1,2,3,4-tetrahydroisoquinoline (1f):

^{1}H-NMR spectrum, ^{13}C-NMR spectrum, COSY spectrum, HSQC spectrum, MS spectrum, HRMS results

Phenylacetyl chloride: ^{1}H-NMR spectrum

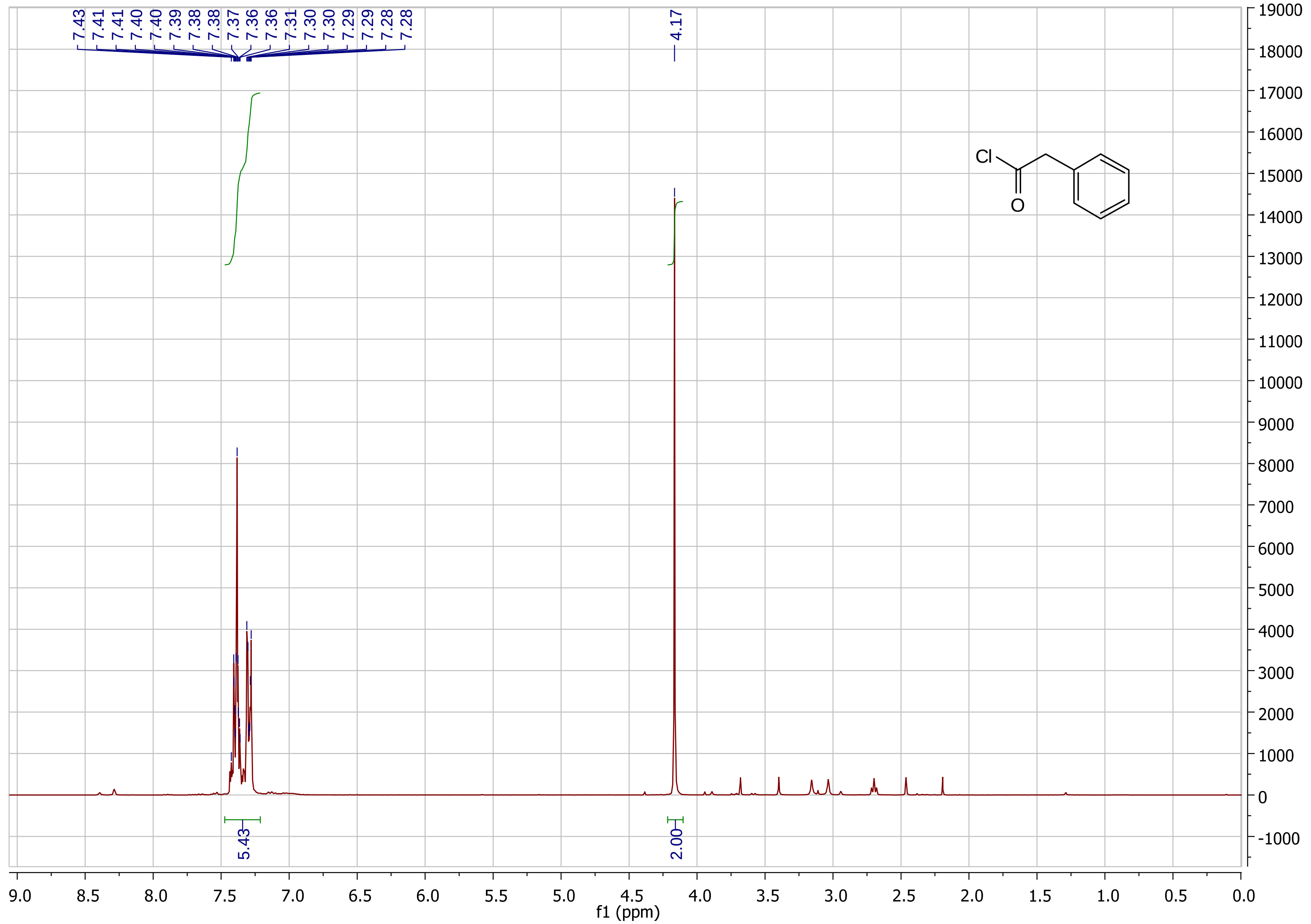

Phenylacetyl chloride: ^{13}C-NMR spectrum

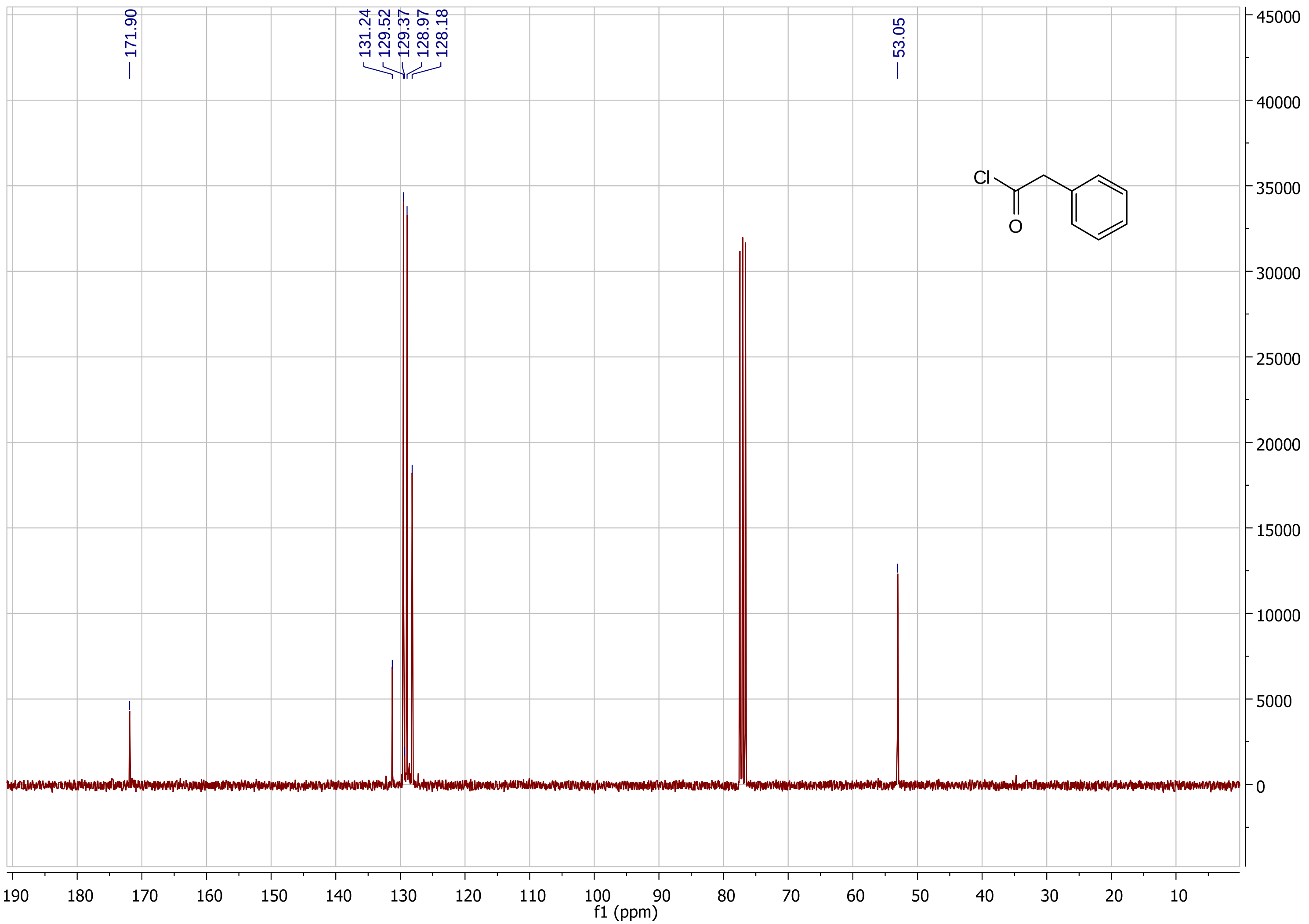

***N*-(3,4-Dimethoxyphenethyl)-*N*-methyl-2-phenylacetamide:** ^{1}H-NMR spectrum

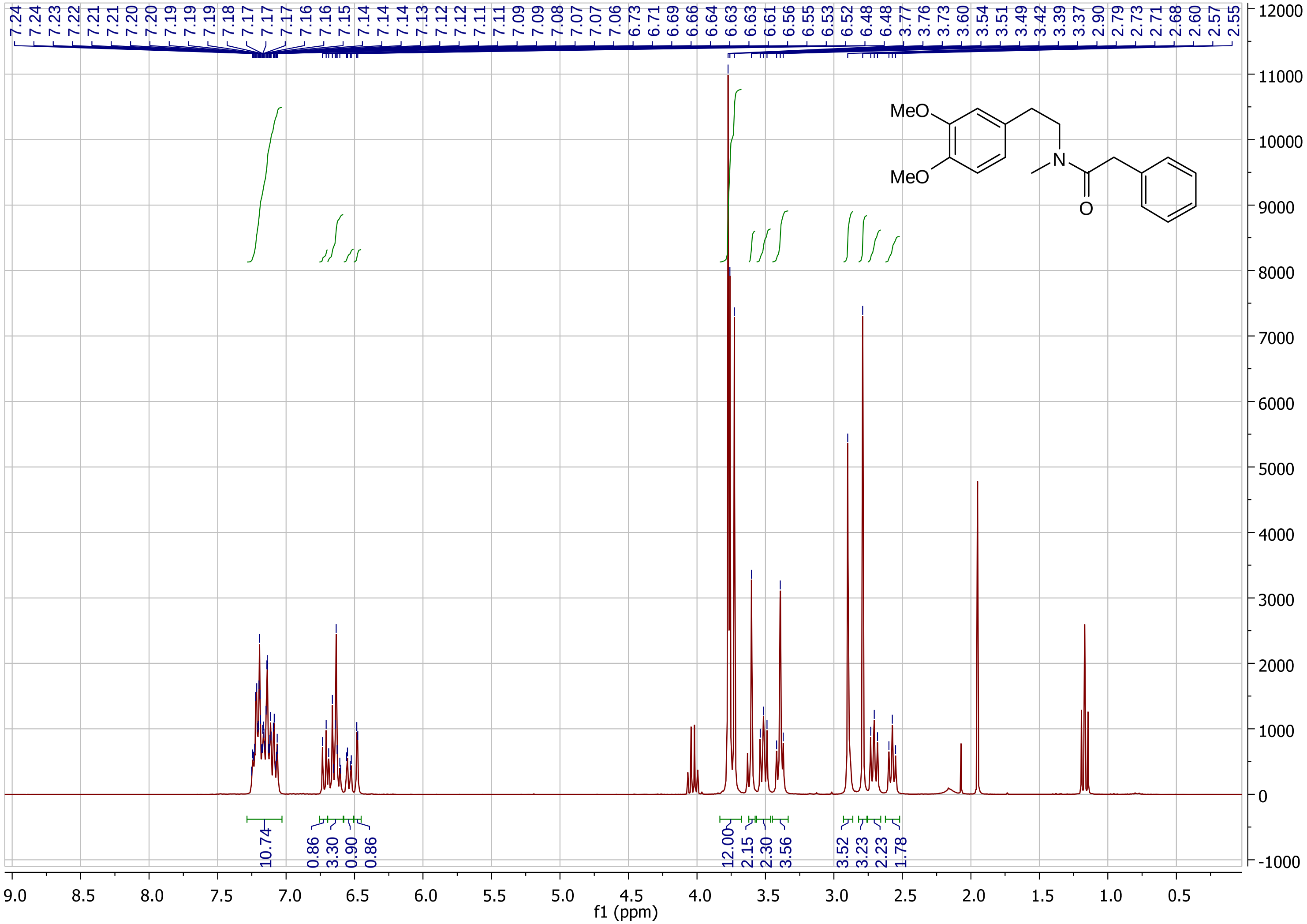

***N*-(3,4-Dimethoxyphenethyl)-*N*-methyl-2-phenylacetamide:** ^{13}C-NMR spectrum

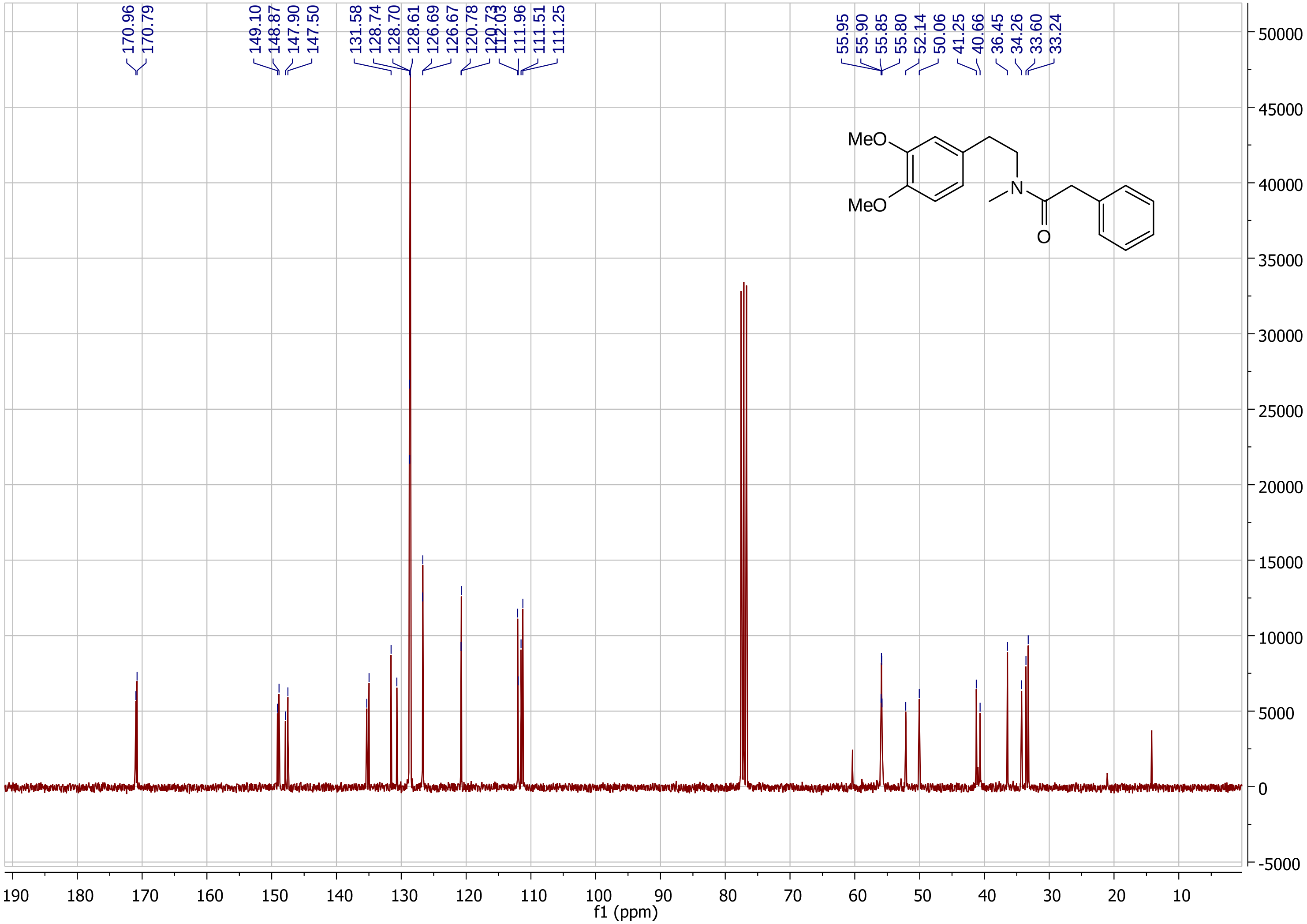

***N*-(3,4-Dimethoxyphenethyl)-*N*-methyl-2-phenylacetamide:** ^{13}C-NMR DEPT135 spectrum

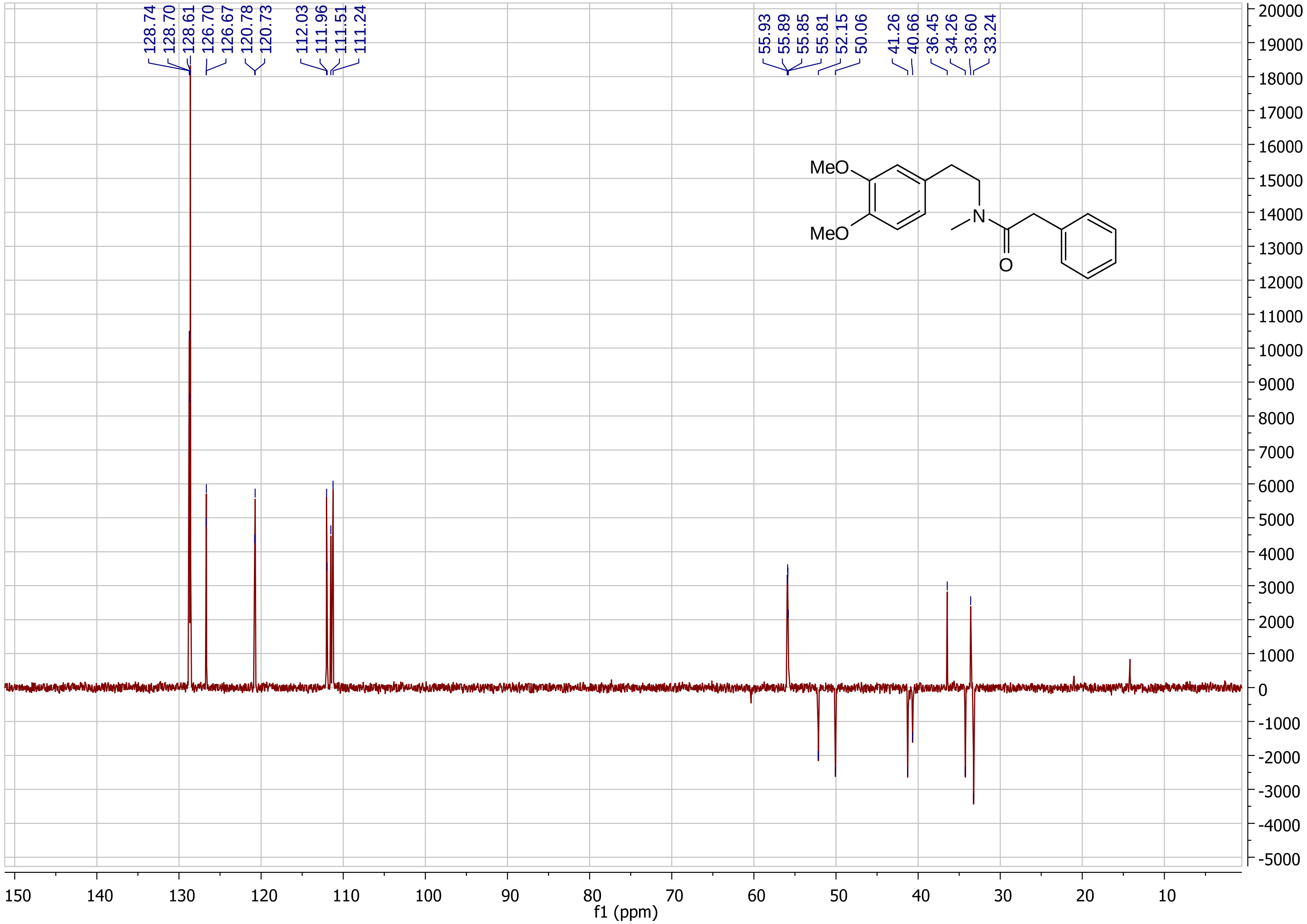

_N_-(3,4-Dimethoxyphenethyl)-_N_-methyl-2-phenylacetamide: ^{13}C-NMR DEPT90 spectrum

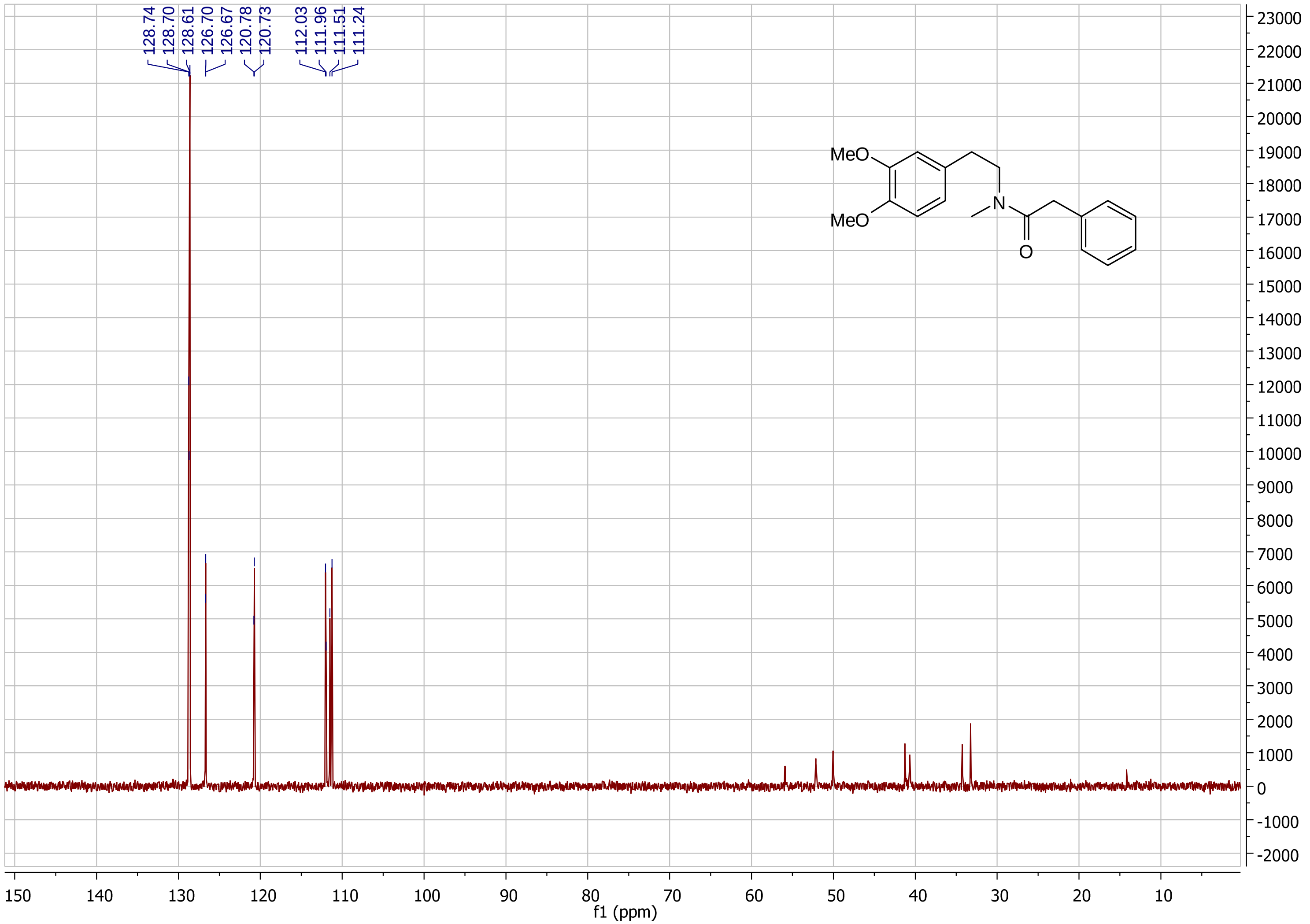

***N*-(3,4-Dimethoxyphenethyl)-*N*-methyl-2-phenylacetamide:** COSY spectrum

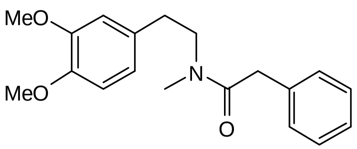

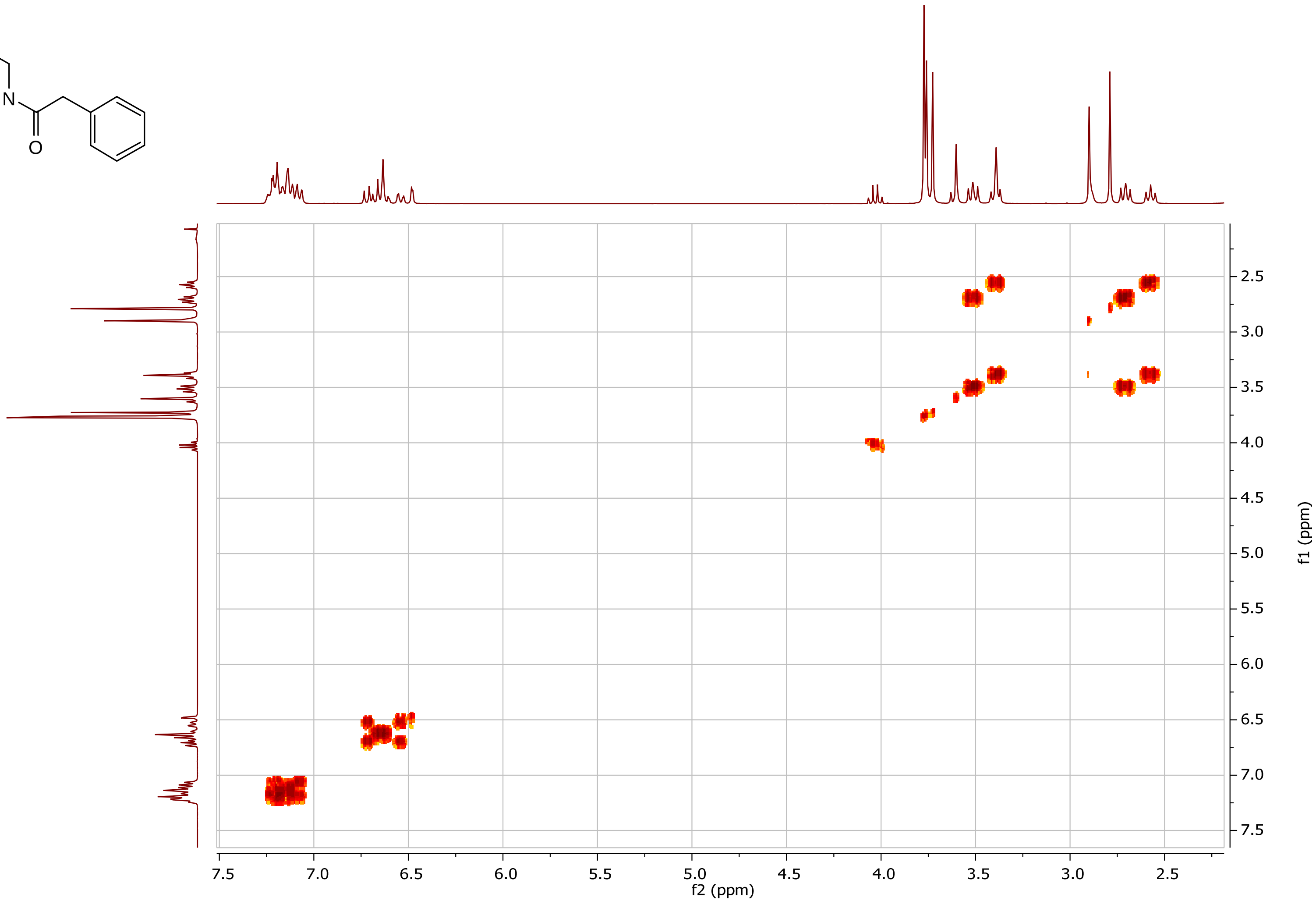

***N*-(3,4-Dimethoxyphenethyl)-*N*-methyl-2-phenylacetamide:** HSQC spectrum

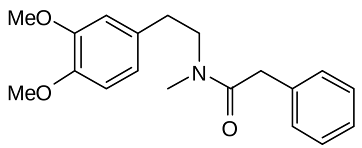

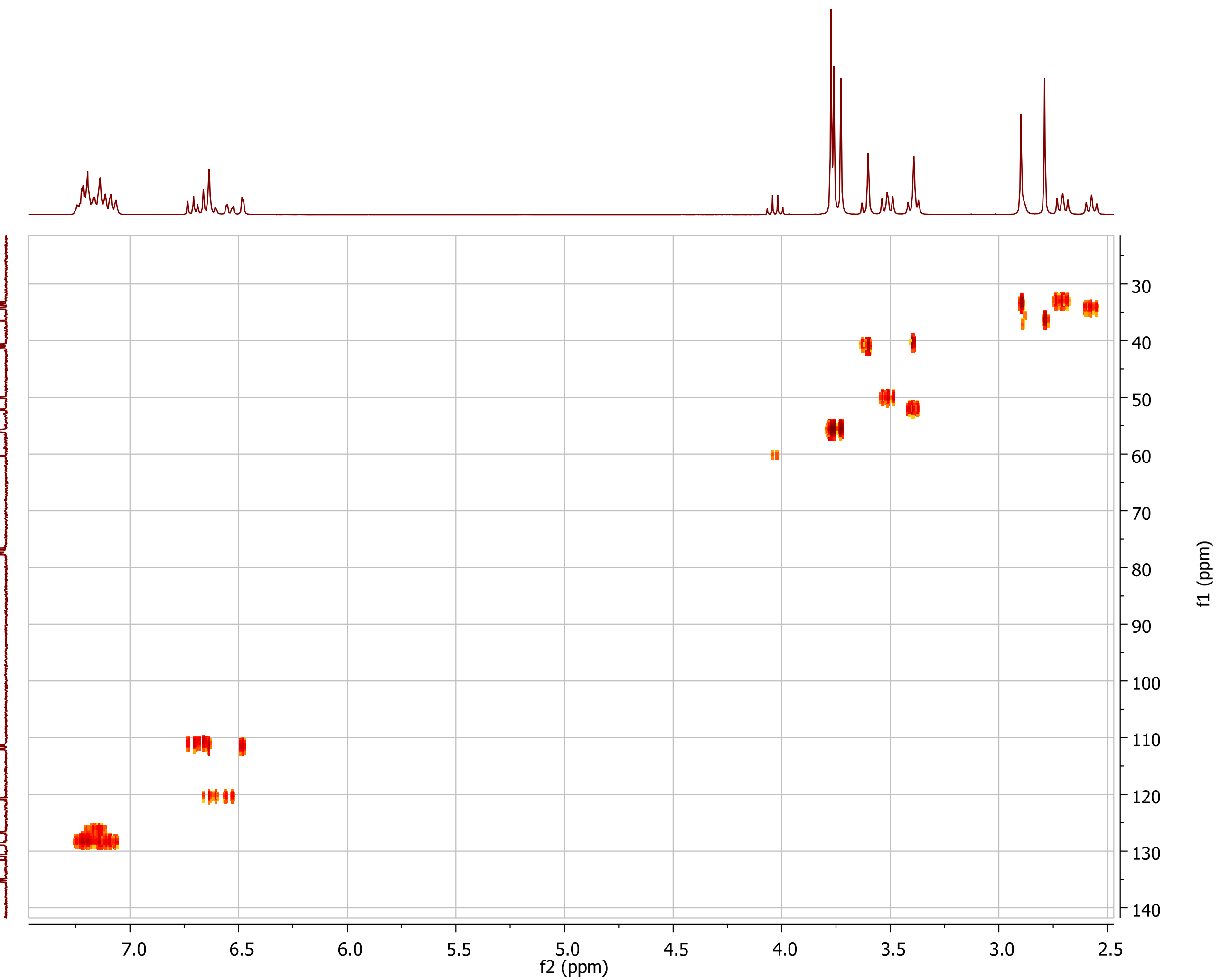

***N*-(3,4-Dimethoxyphenethyl)-*N*-methyl-2-phenylacetamide:** MS spectrum

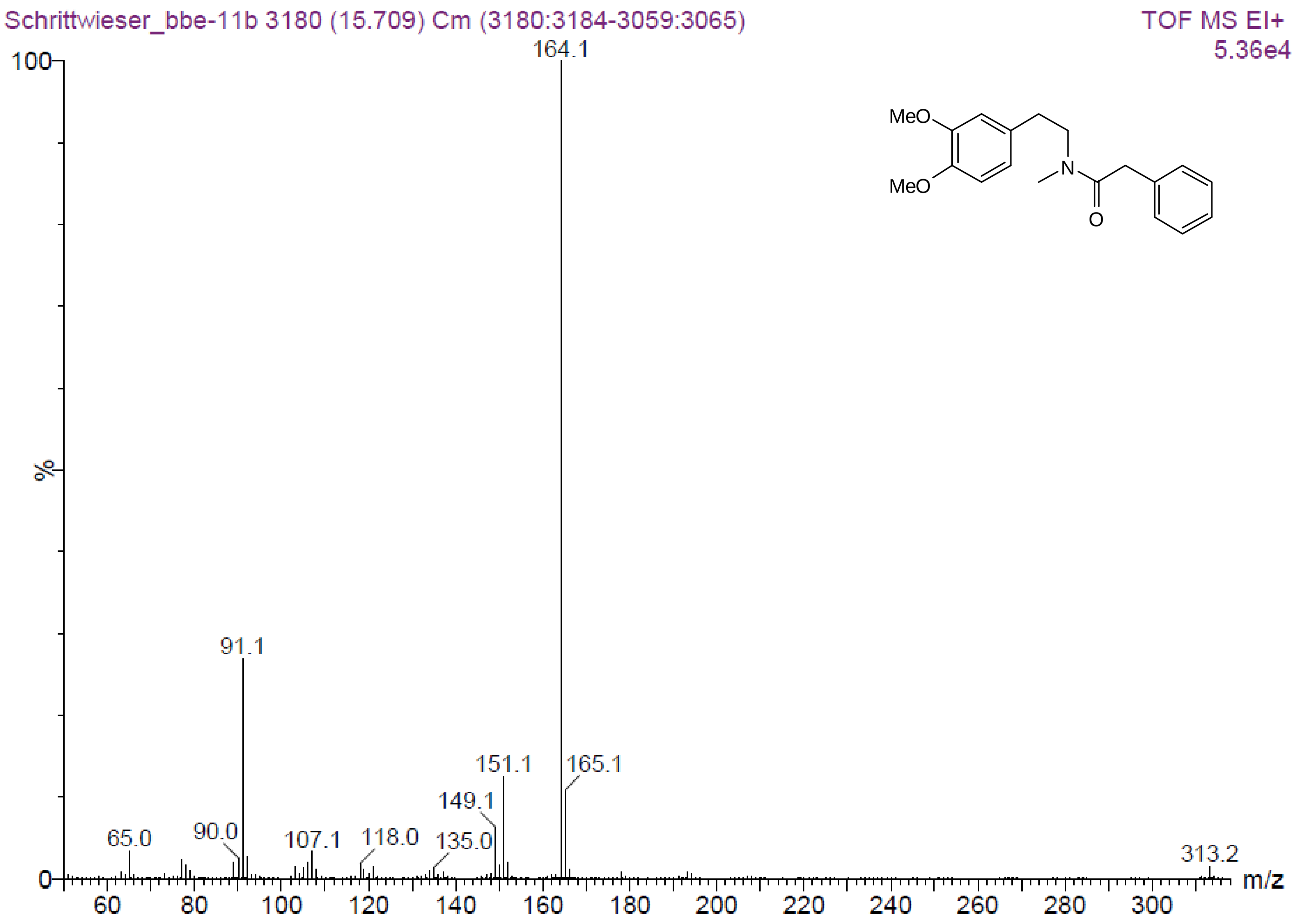

***N*-(3,4-Dimethoxyphenethyl)-*N*-methyl-2-phenylacetamide:** HRMS results

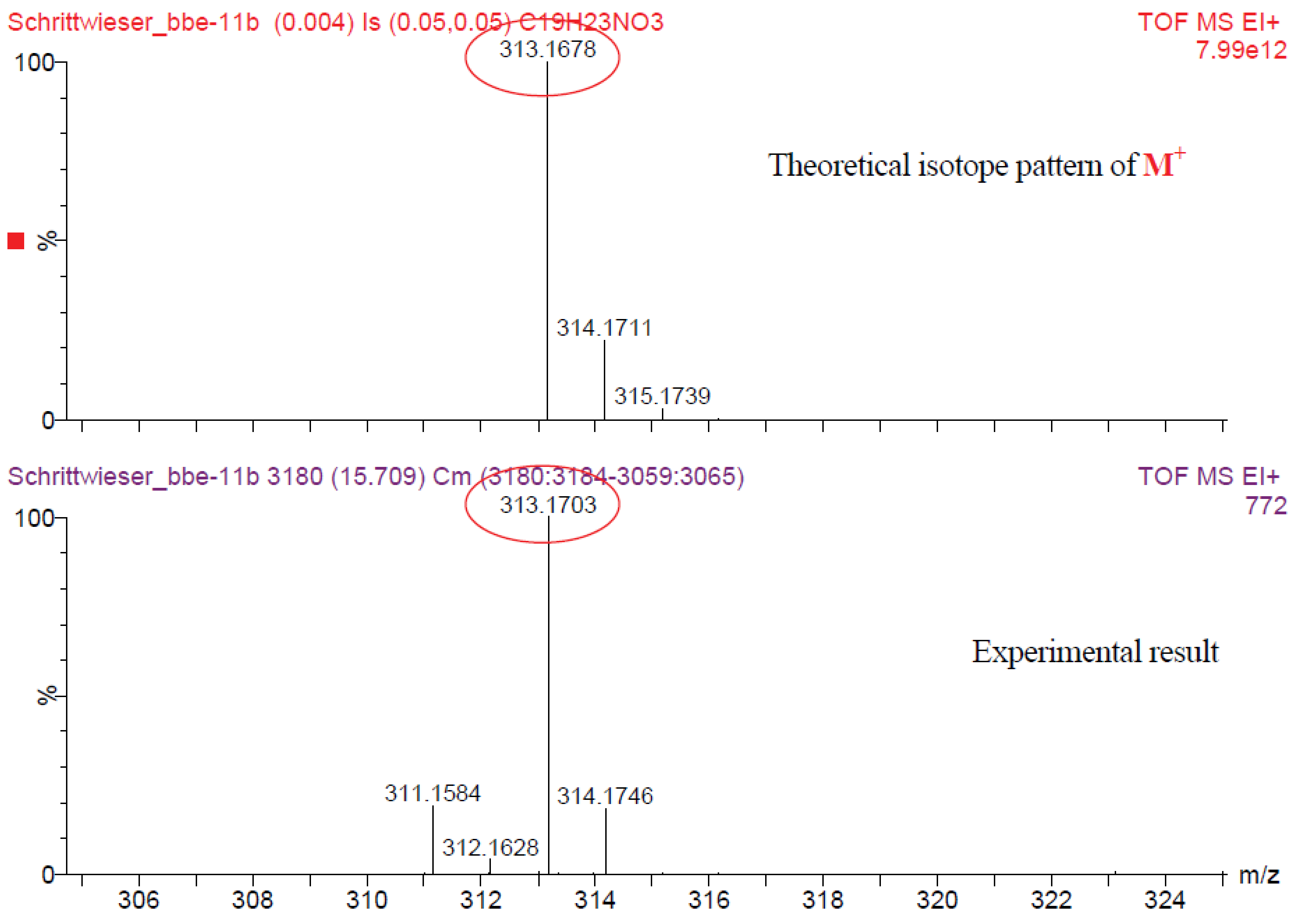

1-Benzyl-6,7-dimethoxy-2-methyl-1,2,3,4-tetrahydroisoquinoline (1f): ^{1}H-NMR spectrum

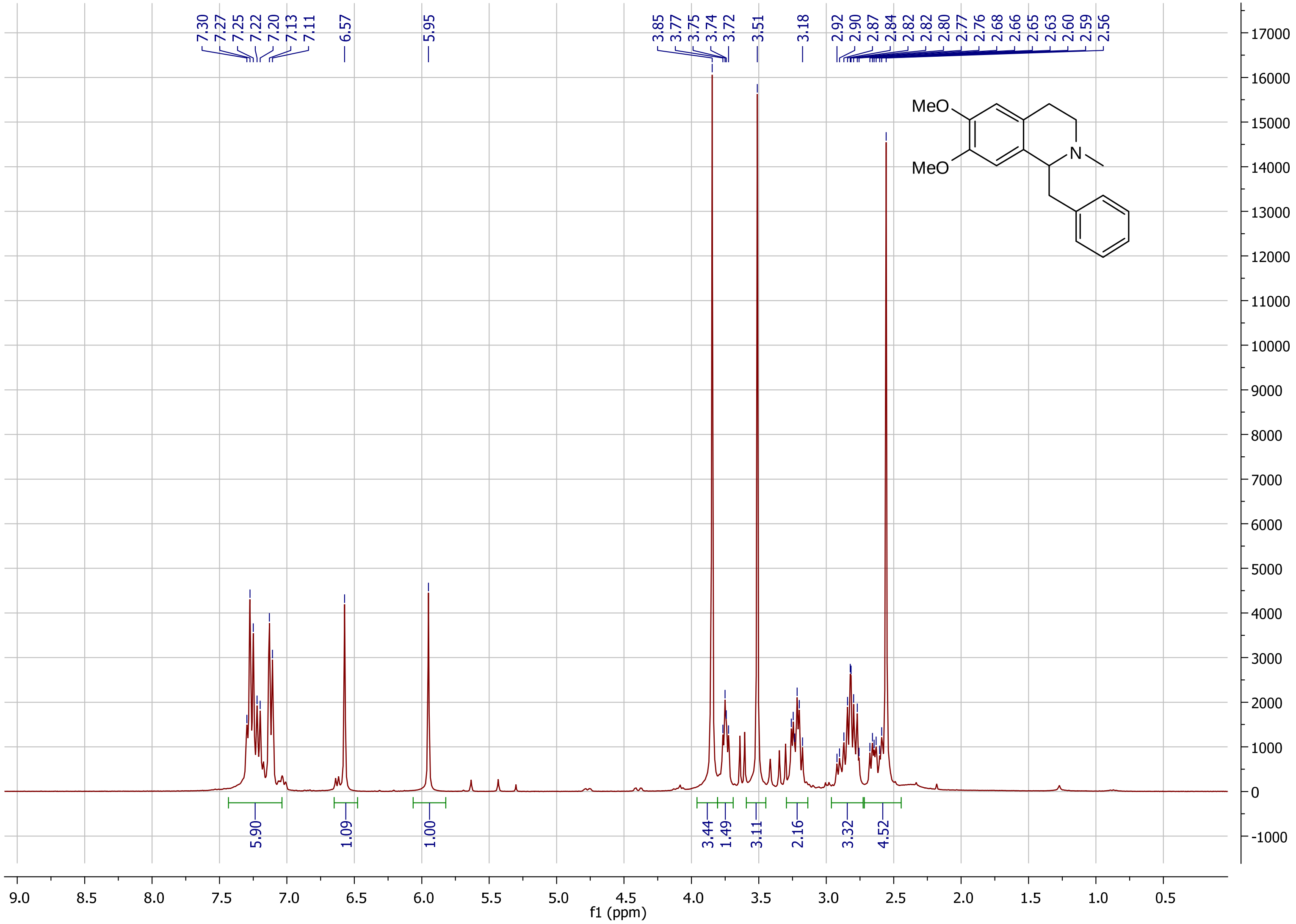

1-Benzyl-6,7-dimethoxy-2-methyl-1,2,3,4-tetrahydroisoquinoline (1f): ^{13}C-NMR spectrum

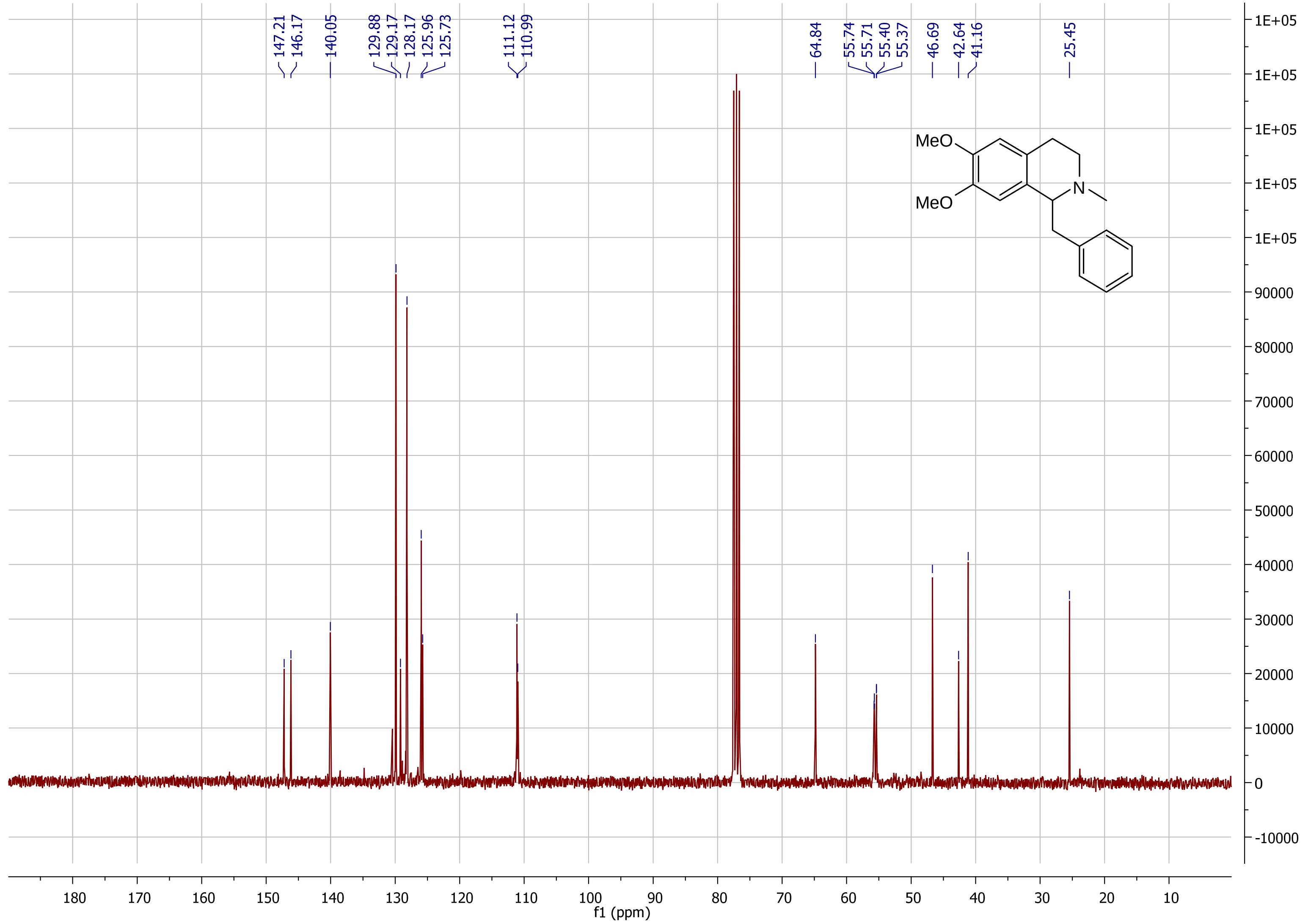

1-Benzyl-6,7-dimethoxy-2-methyl-1,2,3,4-tetrahydroisoquinoline (1f): COSY spectrum

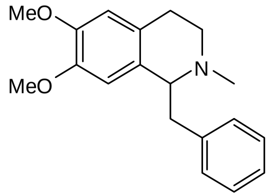

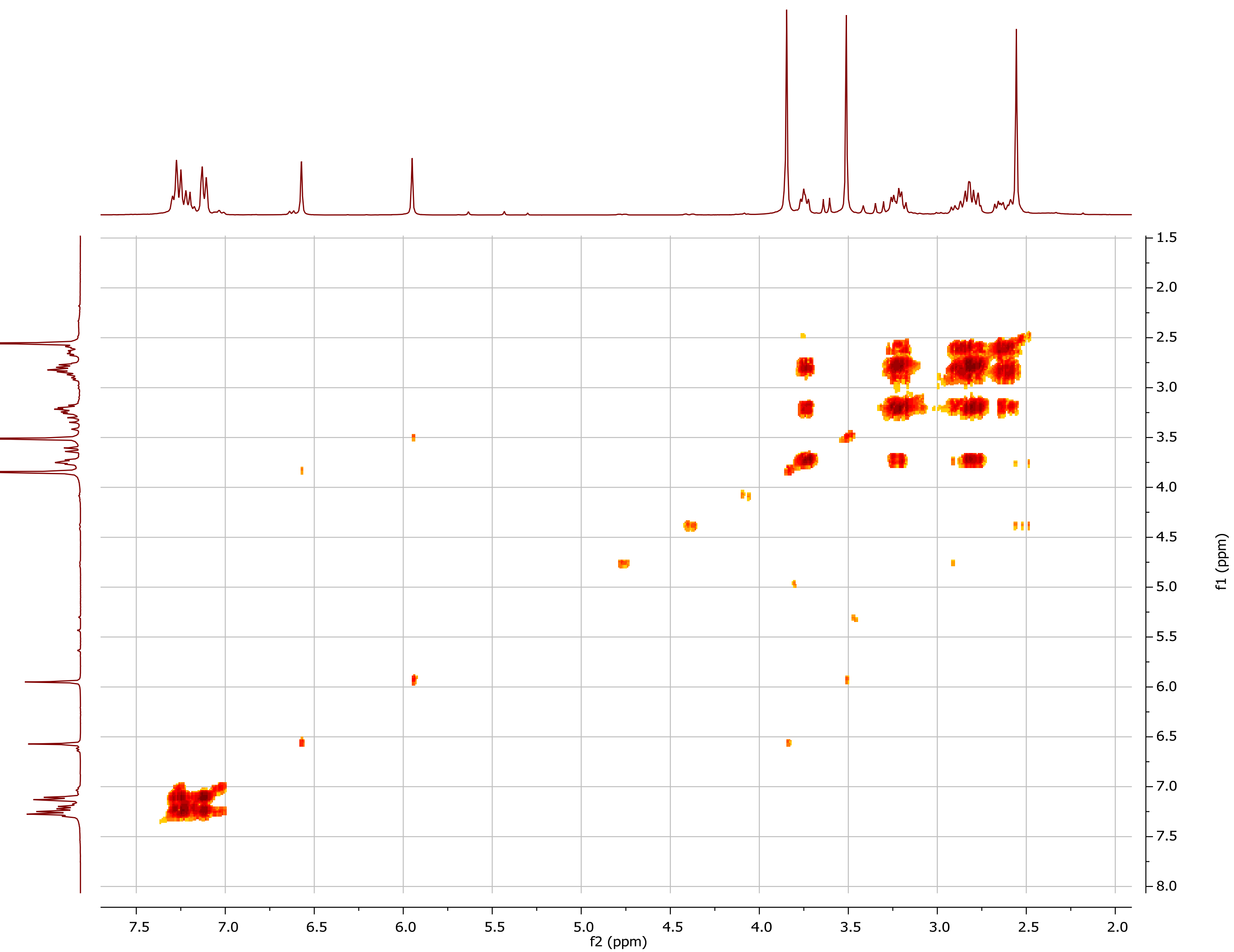

1-Benzyl-6,7-dimethoxy-2-methyl-1,2,3,4-tetrahydroisoquinoline (1f): HSQC spectrum

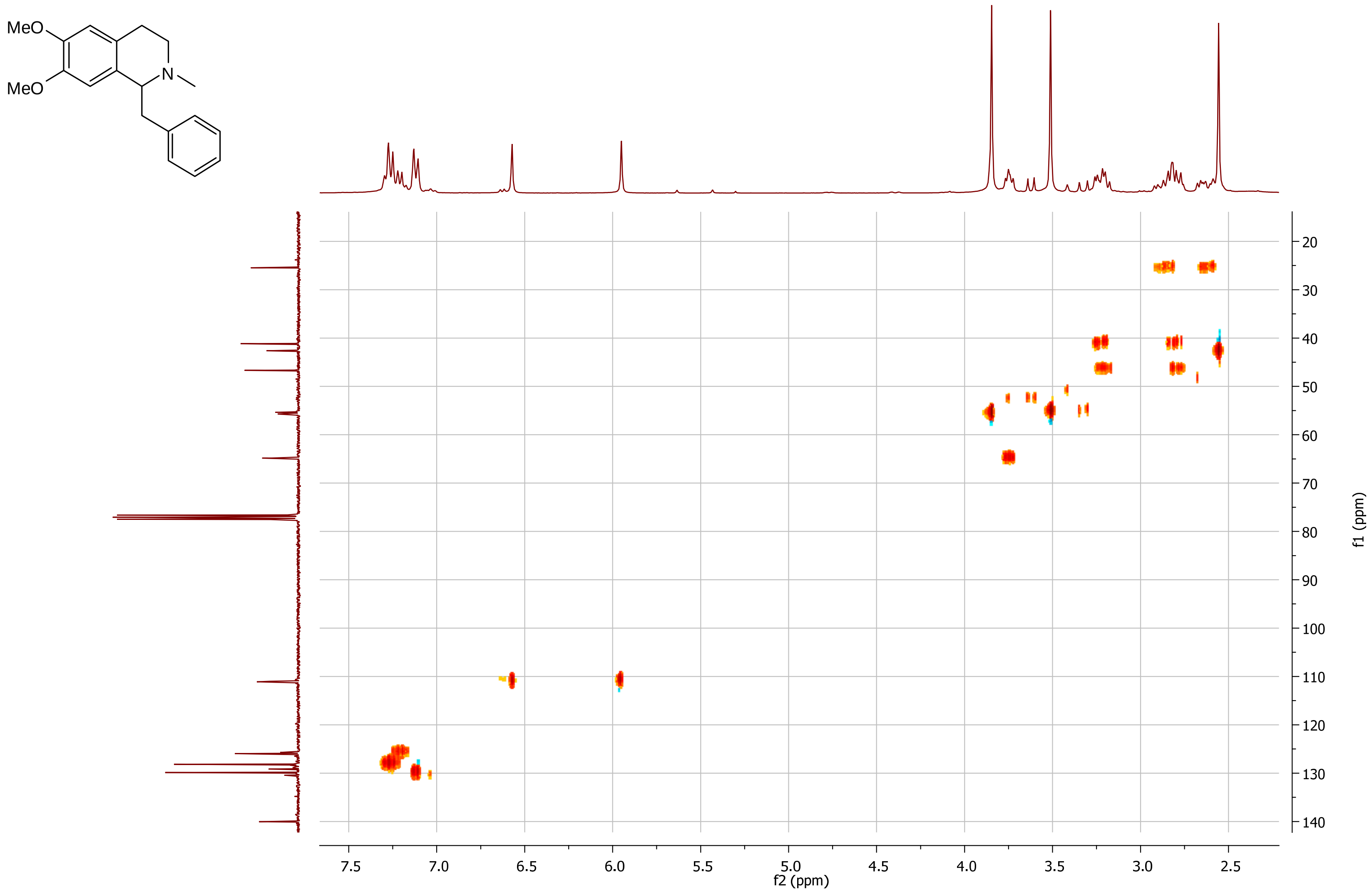

1-Benzyl-6,7-dimethoxy-2-methyl-1,2,3,4-tetrahydroisoquinoline (1f): MS spectrum

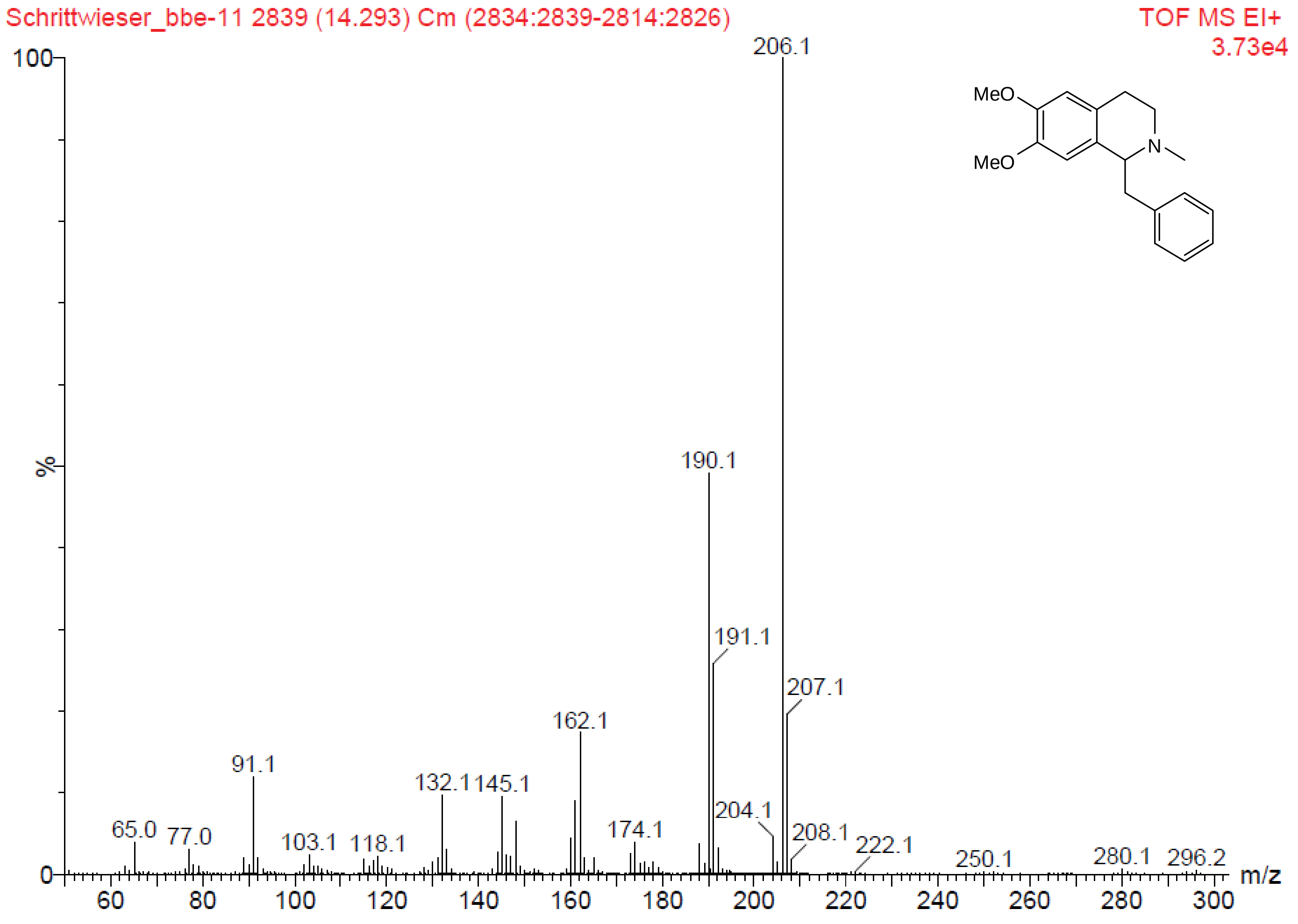

1-Benzyl-6,7-dimethoxy-2-methyl-1,2,3,4-tetrahydroisoquinoline (1f): HRMS results

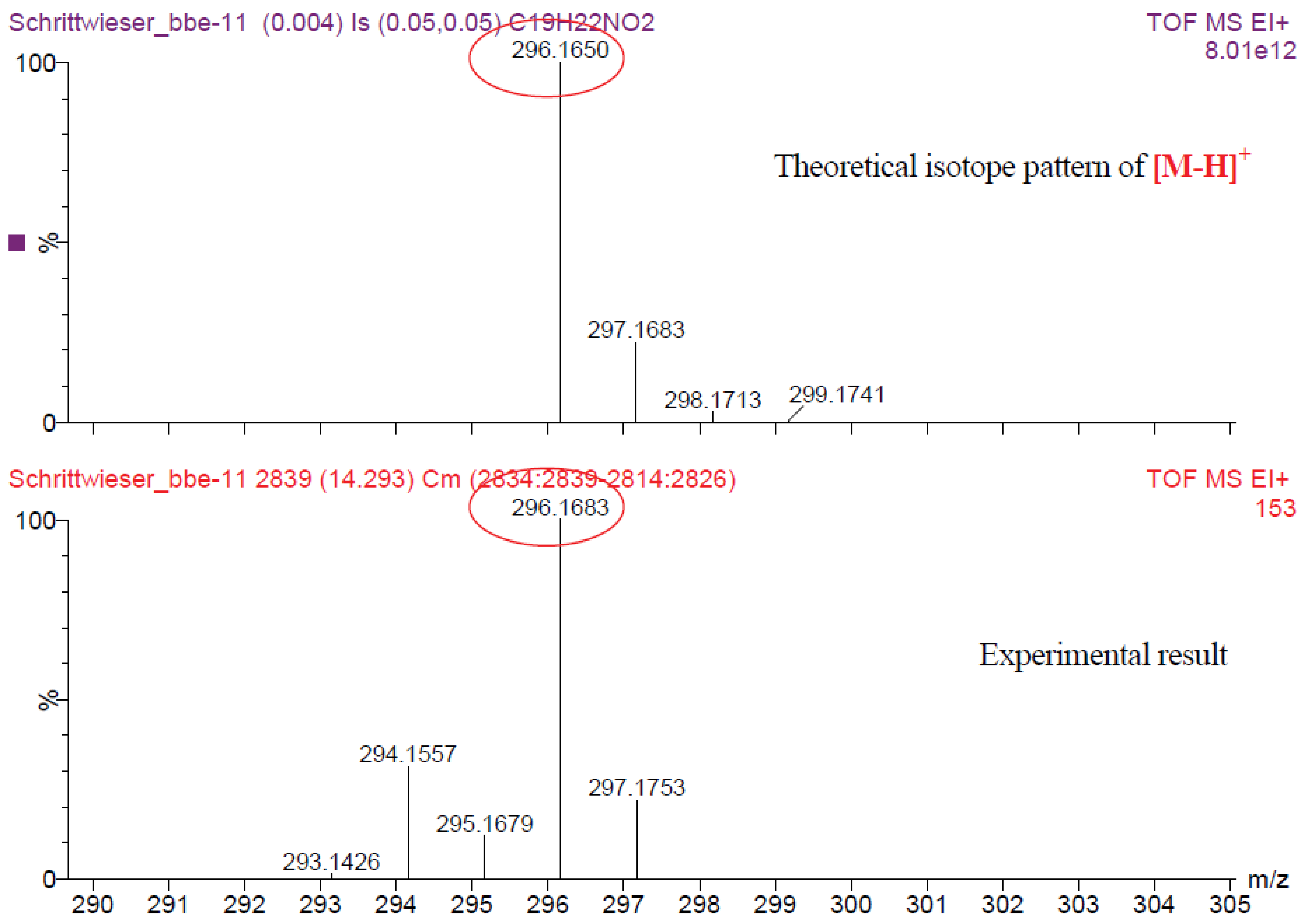

Products of Enzymatic Conversions

Provided Material:

(*S*)-2,9-Dihydroxy-3-methoxyberbine (*S*)-2a:

^{1}H-NMR spectrum, ^{13}C-NMR spectrum, HSQC spectrum, MS spectrum, chiral-phase HPLC chromatogram

(*S*)-Scoulerine (*S*)-2b:

^{1}H-NMR spectrum, ^{13}C-NMR spectrum, HSQC spectrum, MS spectrum, chiral-phase HPLC chromatogram

(*S*)-2,9-Dihydroxy-3-methoxyberbine (*S*)-2a: ^{1}H-NMR spectrum

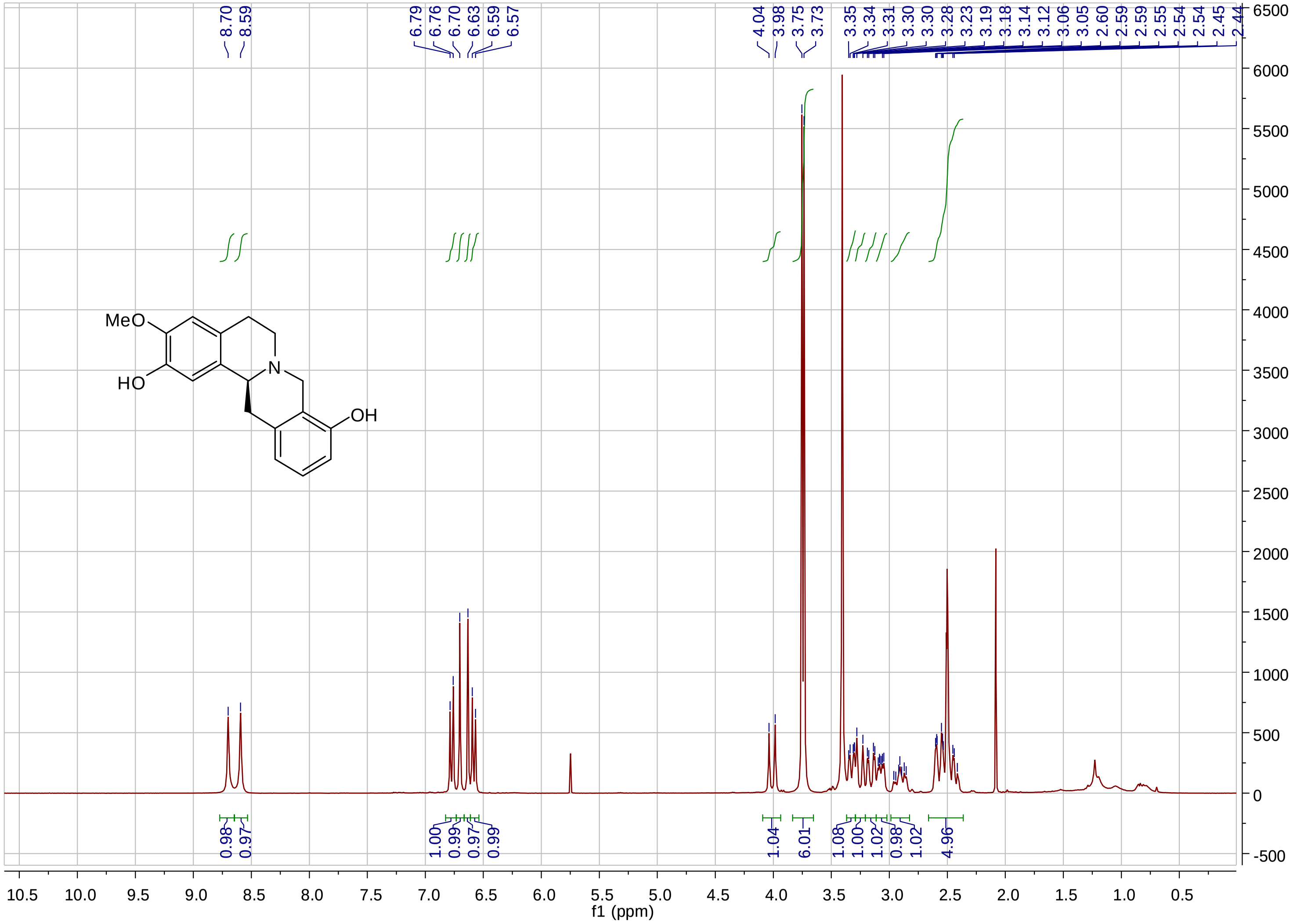

(*S*)-2,9-Dihydroxy-3-methoxyberbine (*S*)-2a: ^{13}C-NMR spectrum

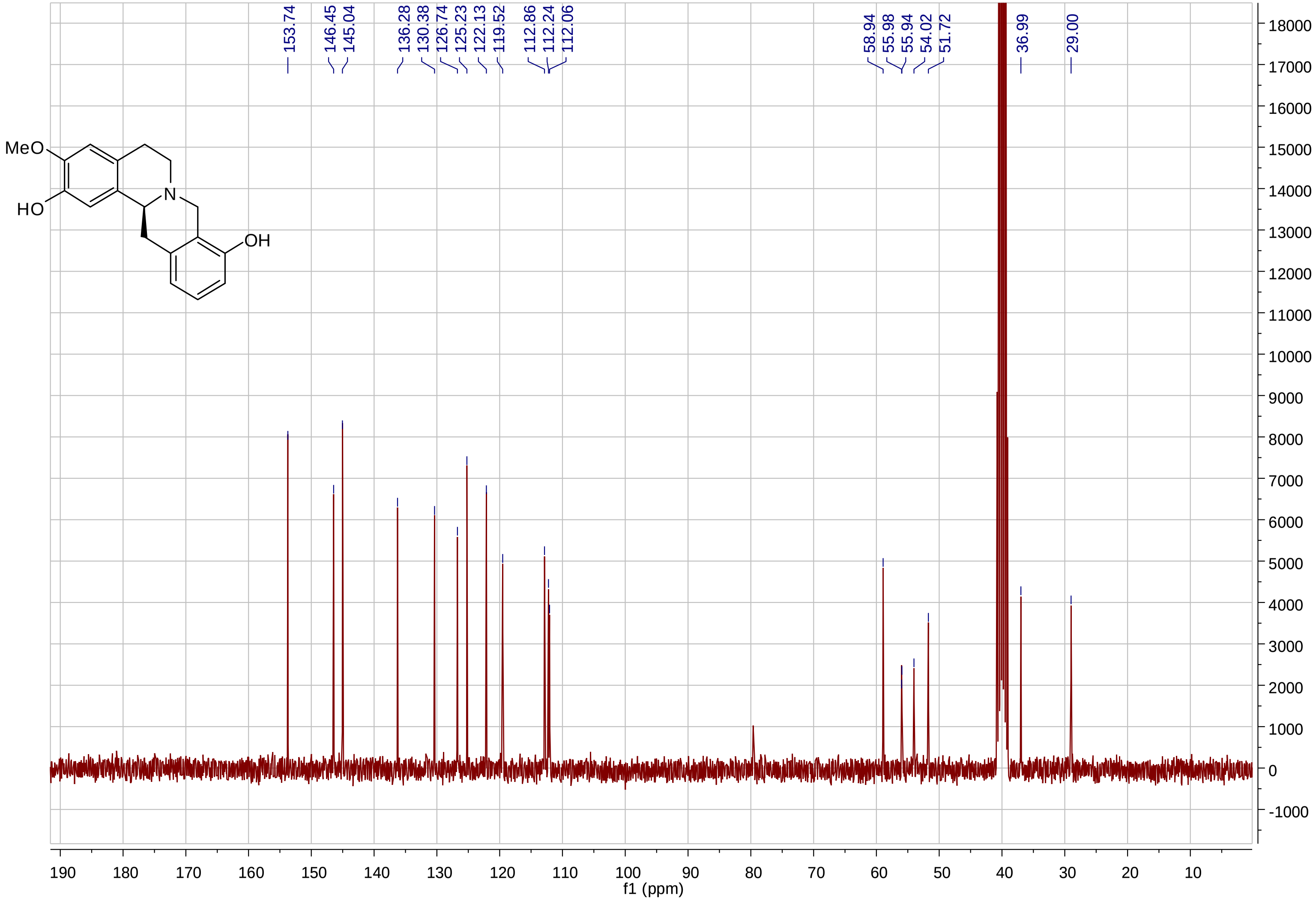

(*S*)-2,9-Dihydroxy-3-methoxyberbine (*S*)-2a: HSQC spectrum

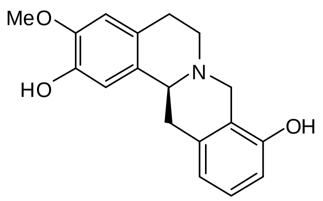

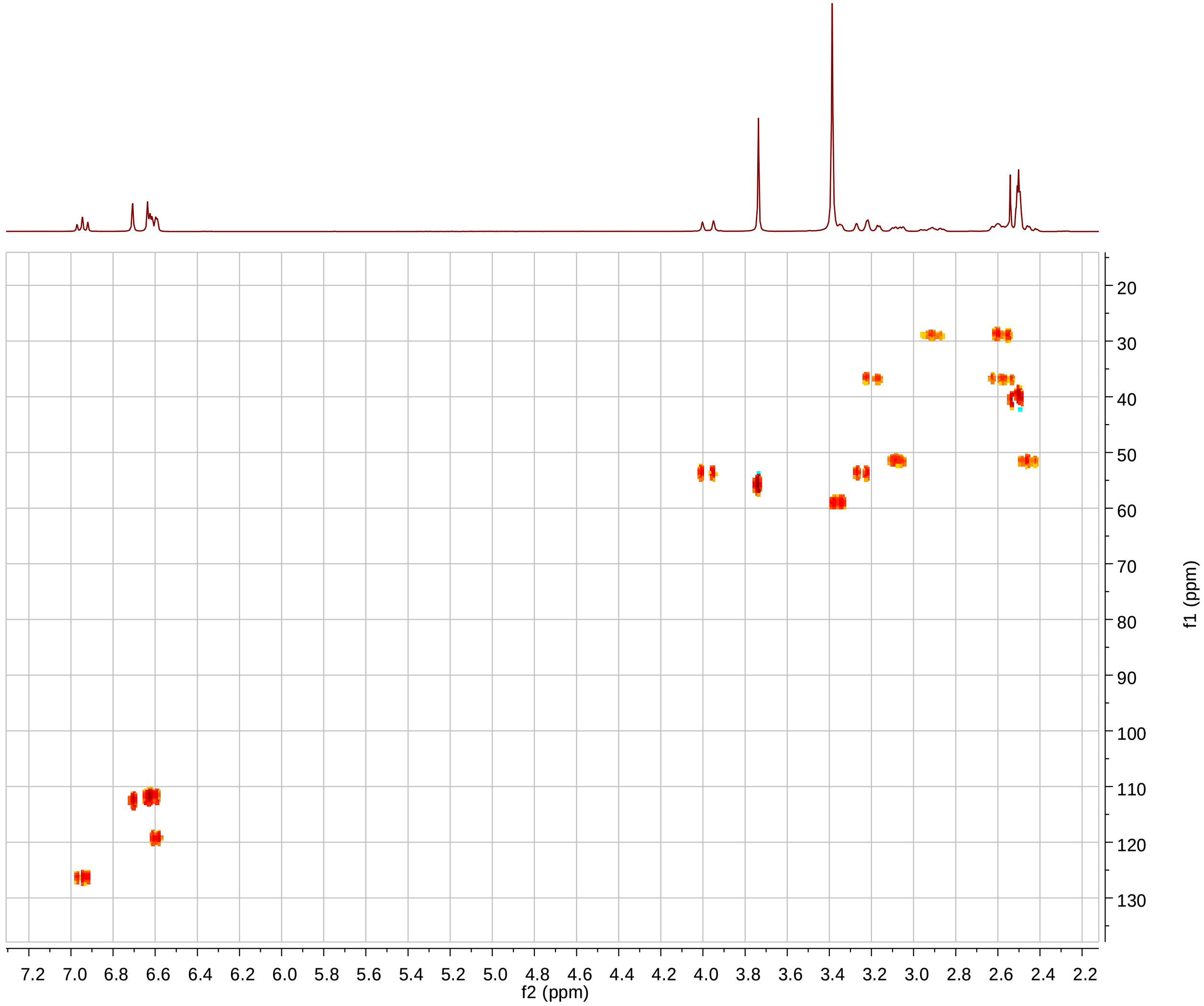

(*S*)-2,9-Dihydroxy-3-methoxyberbine (*S*)-2a: MS spectrum

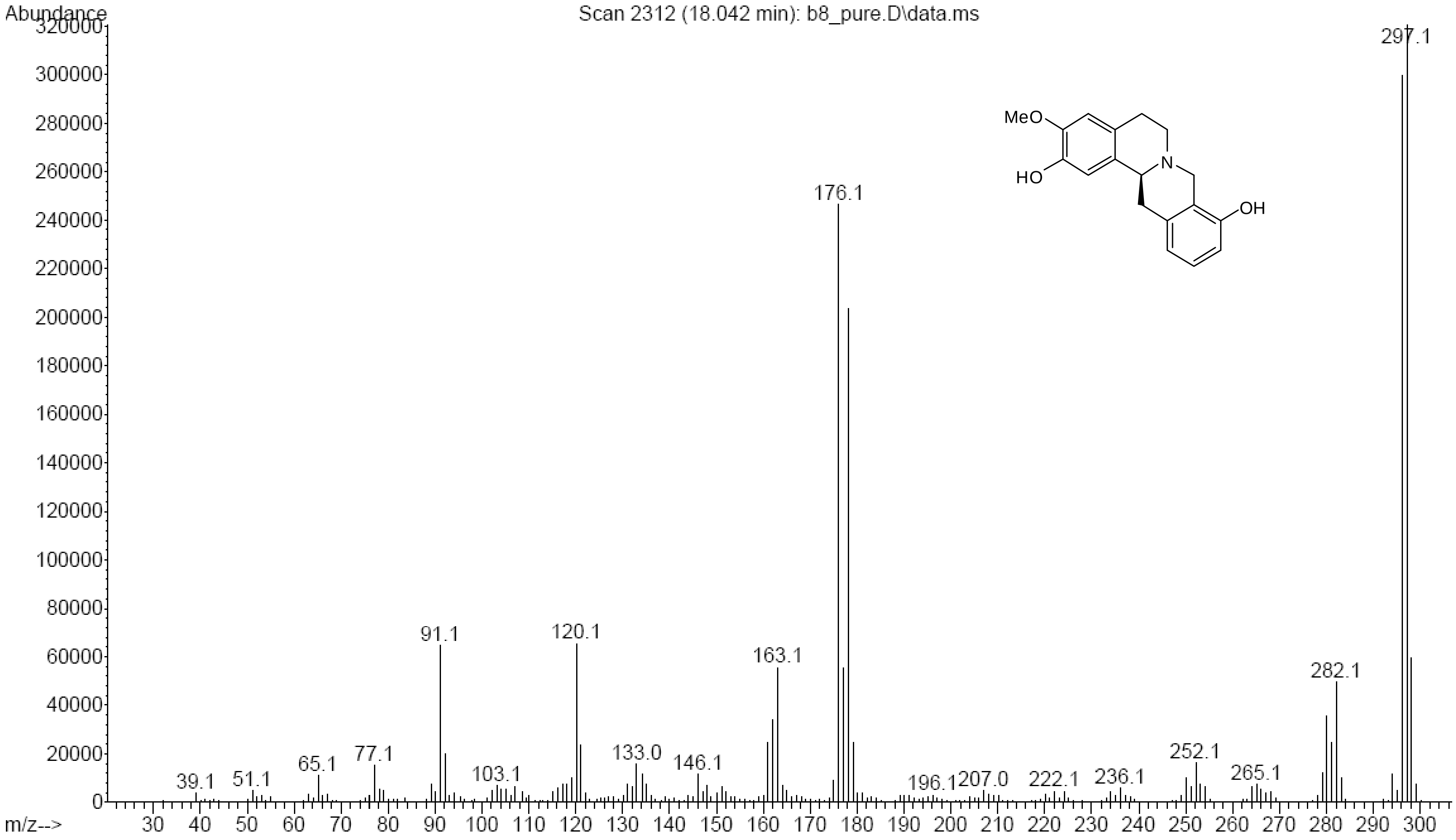

(*S*)-2,9-Dihydroxy-3-methoxyberbine (*S*)-2a: Chiral-phase HPLC chromatogram

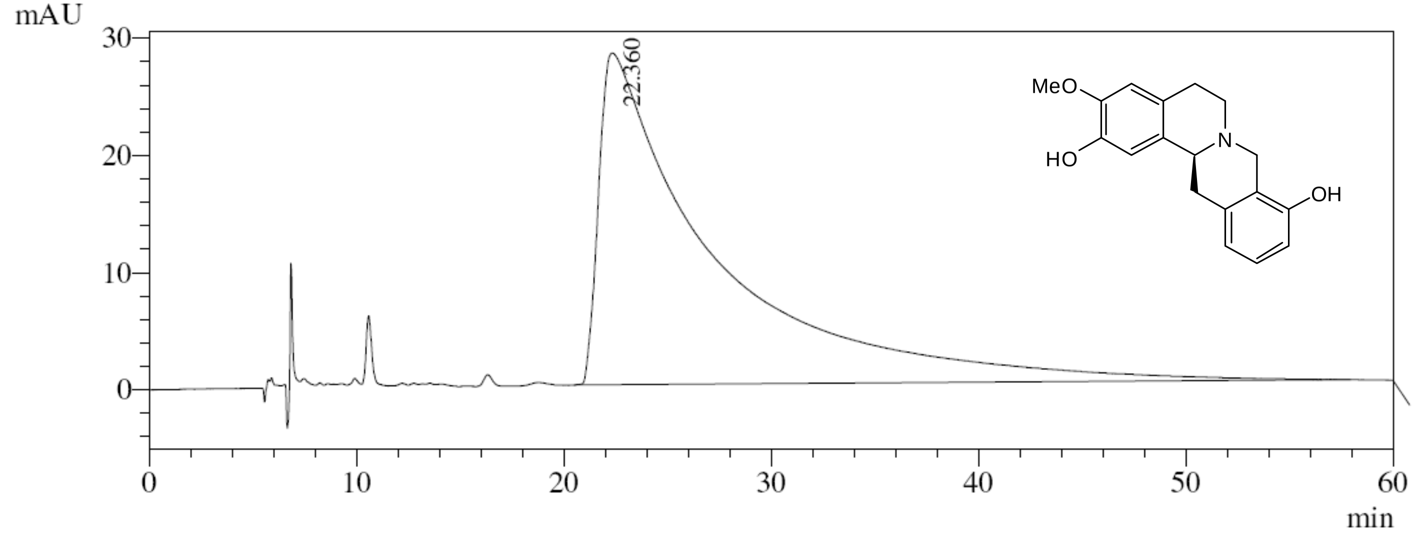

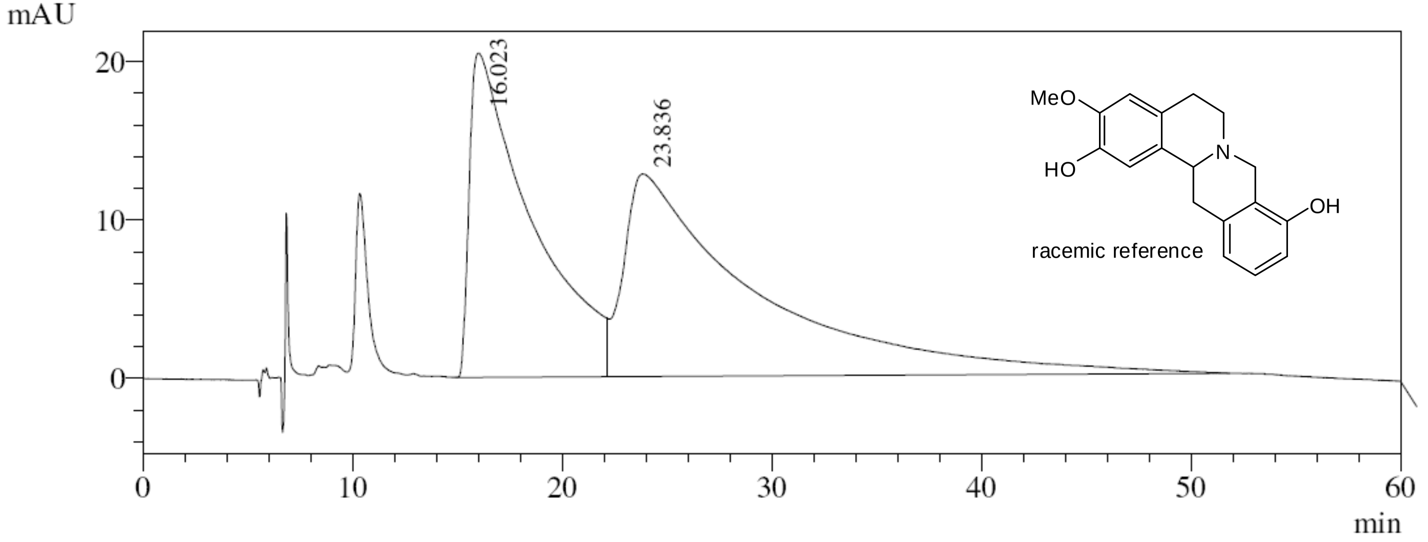

(*S*)-Scoulerine (*S*)-2b: ^{1}H-NMR spectrum

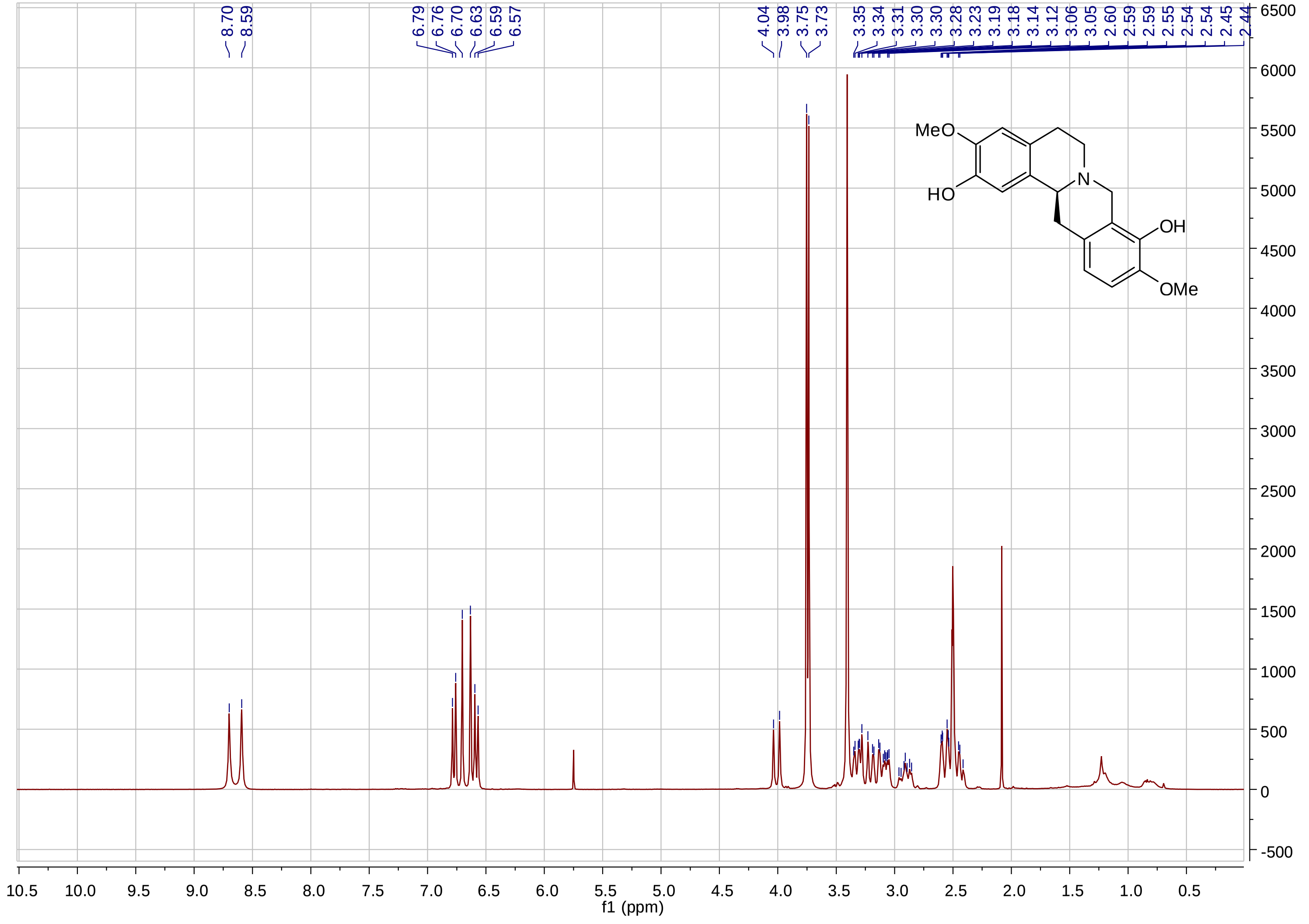

(*S*)-Scoulerine (*S*)-2b: ^{13}C-NMR spectrum

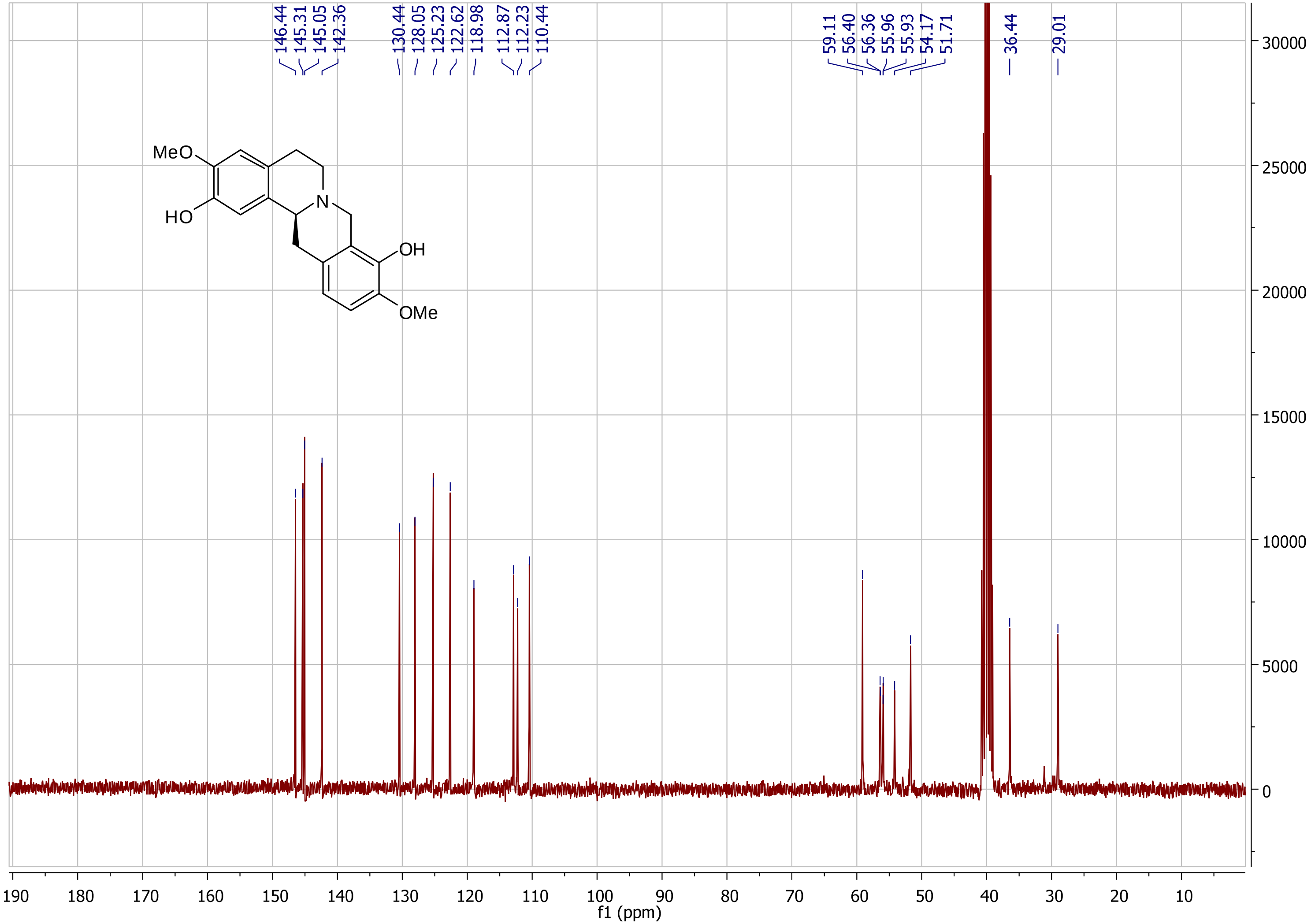

(*S*)-Scoulerine (*S*)-2b: HSQC spectrum

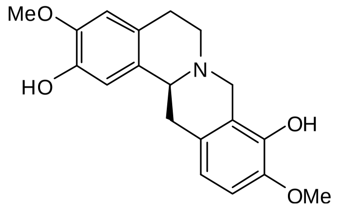

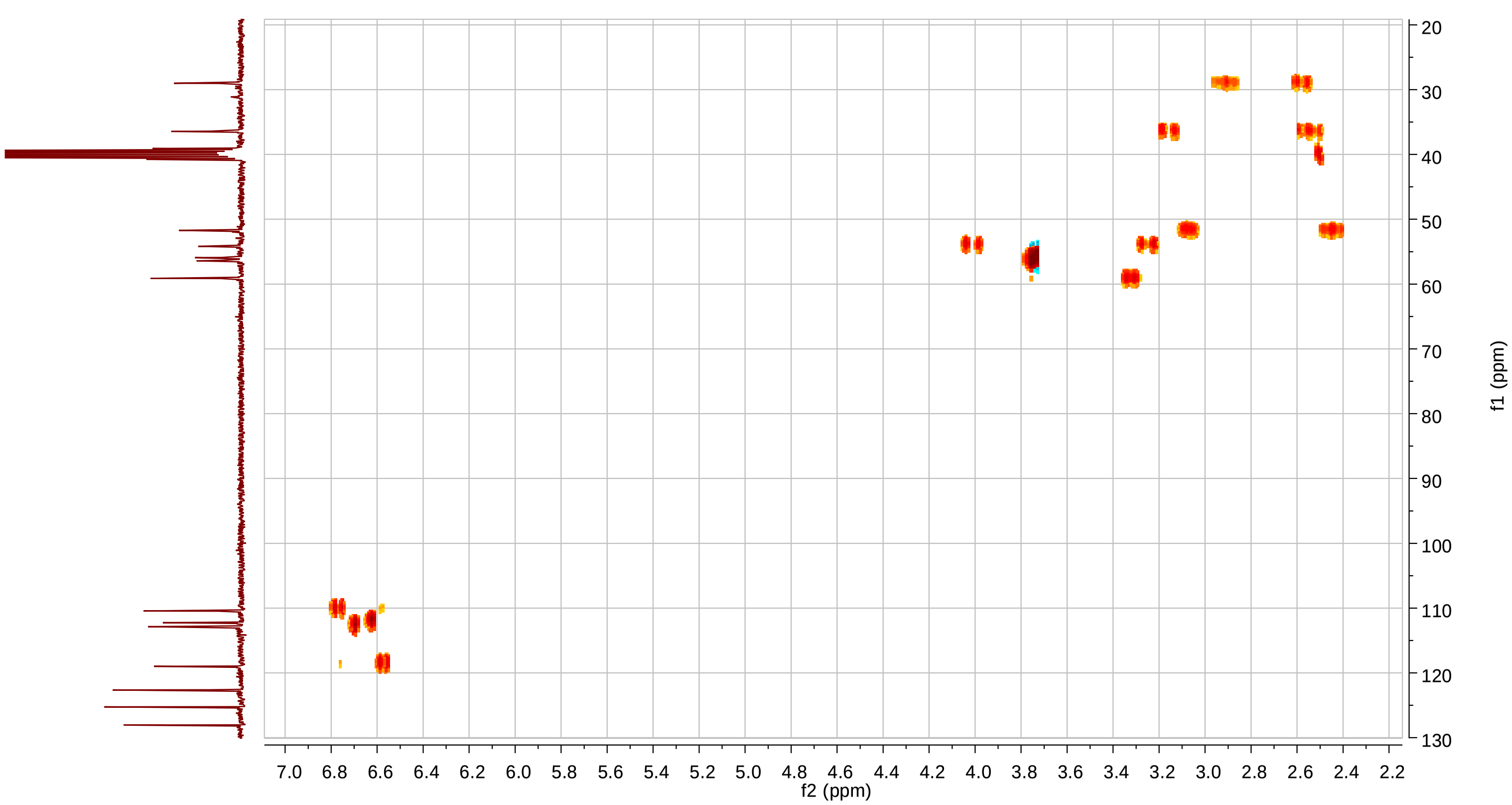

(*S*)-Scoulerine (*S*)-2b: MS spectrum

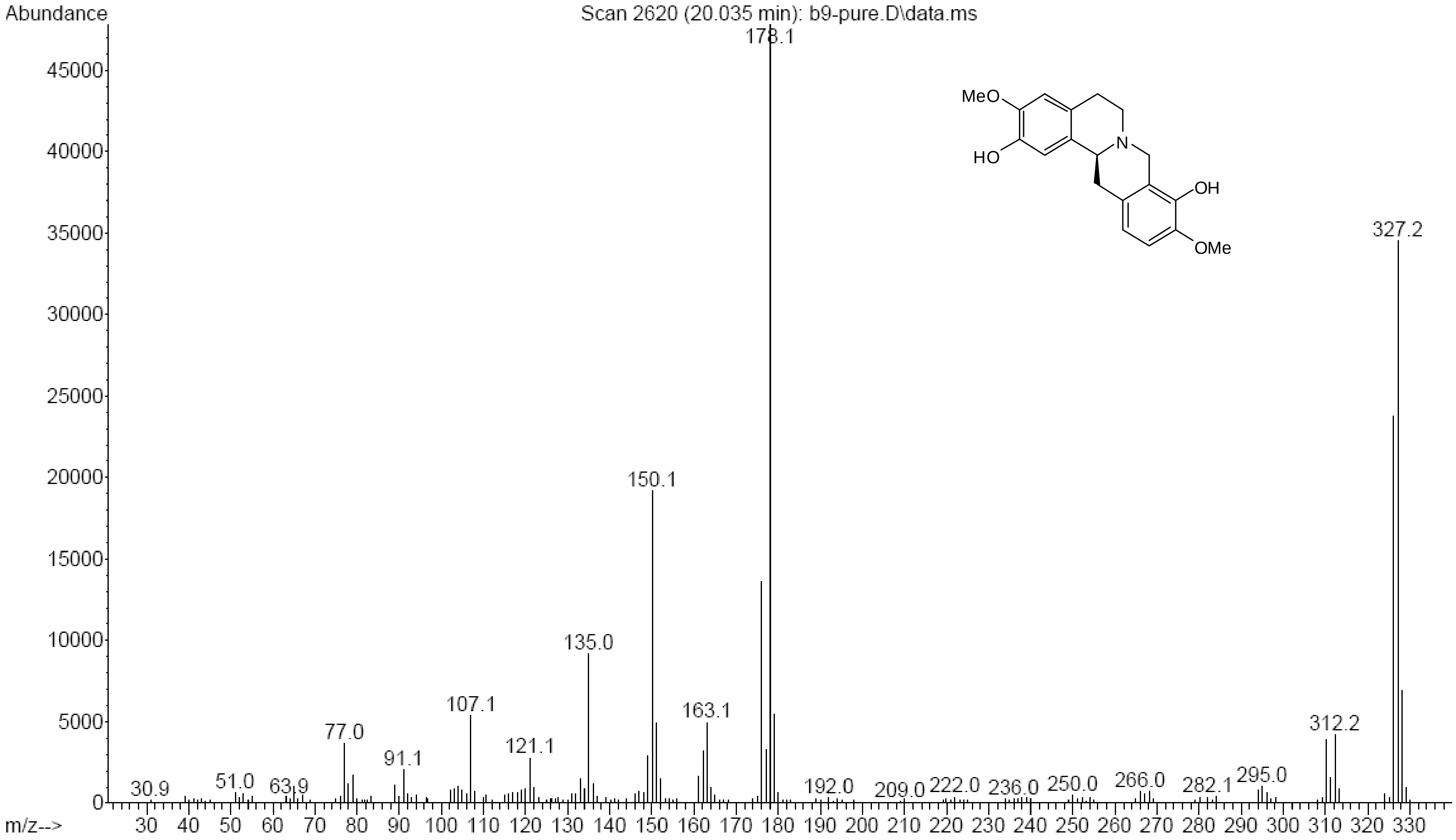

(*S*)-Scoulerine (*S*)-2b: Chiral-phase HPLC chromatogram

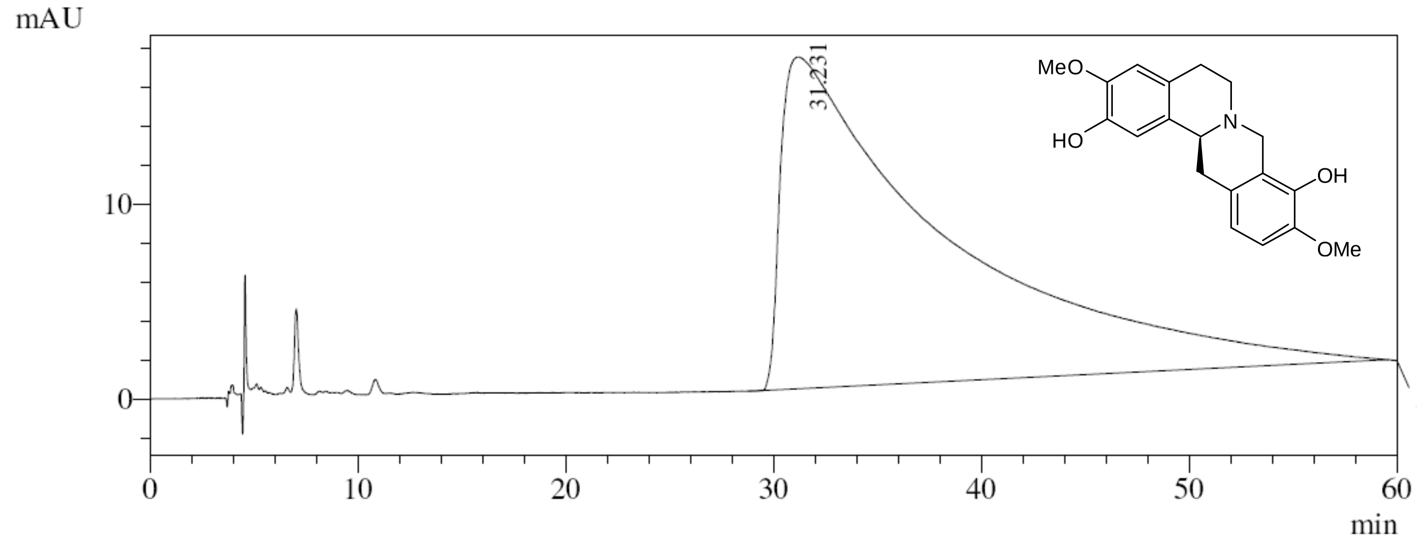

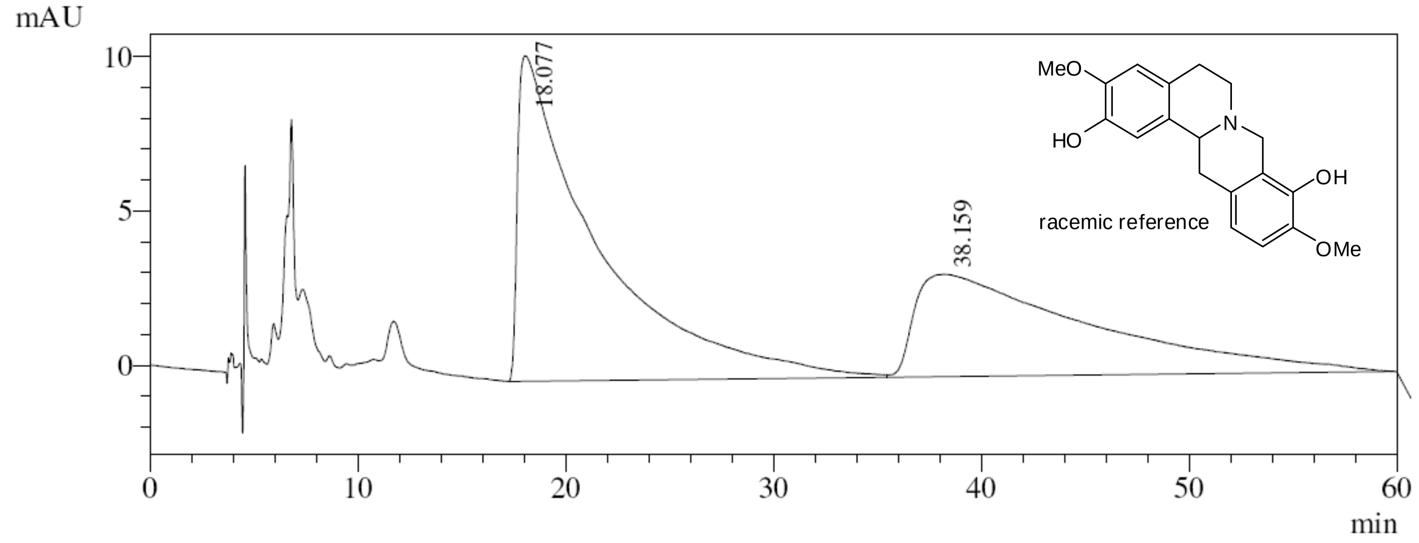

References:

[1] Chen, C.-S.; Fujimoto, Y.; Girdaukas, G.; Sih, C. J. *J. Am. Chem. Soc.* **1982**, *104*, 7294–7299.
[2] In view of the proposed mechanism for MAOs, the oxidation product is most likely the corresponding 3,4-dihydroisoquinolinium ion. See: D. E. Edmondson, A. Mattevi, C. Binda, M. Li, F. Hubálek, *Curr. Med. Chem.* **2004**, *11*, 1983–1993. However, this species can interconvert with the corresponding enamine in basic aqueous solution. See: X.-S. He, *J. Nat. Prod.* **1993**, *56*, 973–975.
[3] a) J. H. Schrittwieser, V. Resch, J. H. Sattler, W.-D. Lienhart, K. Durchschein, A. Winkler, K. Gruber, P. Macheroux, W. Kroutil, *Angew. Chem.* **2011**, *123*, 1100–1103; *Angew. Chem. Int. Ed.* **2011**, *50*, 1068–1071; b) Schrittwieser, J. H.; Resch, V.; Wallner, S.; Lienhart, W.-D.; Sattler, J. H.; Resch, J.; Macheroux, P.; Kroutil, W. *J. Org. Chem.* **2011**, *76*, 6703–6714.
[4] Damkaci, F.; DeShong, P. *J. Am. Chem. Soc.* **2003**, *125*, 4408–4409.
[5] Kametani, T.; Wakisaka, K.; Fukumoto, K. *Yakugaku Zasshi* **1965**, *85*, 956–959.
[6] Shigehisa, H.; Honda, T. *Heterocycles* **2008**, *75*, 1233–1239.
[7] Resch, V.; Schrittwieser, J. H.; Wallner, S.; Macheroux, P.; Kroutil, W. *Adv. Synth. Catal.* **2011**, *353*, 2377–2383.
